# Stepwise Size Shrinkage Cascade‐Activated Supramolecular Prodrug Boosts Antitumor Immunity by Eliciting Pyroptosis

**DOI:** 10.1002/advs.202203353

**Published:** 2022-07-22

**Authors:** Meng‐Yun Liang, Meng‐Jie Zhang, Wei Qiu, Yao Xiao, Meng‐Jie Ye, Peng Xue, Yue‐Jun Kang, Zhi‐Jun Sun, Zhigang Xu

**Affiliations:** ^1^ State Key Laboratory of Silkworm Genome Biology School of Materials and Energy and Chongqing Key Laboratory of Soft‐Matter Material Chemistry and Function Manufacturing Southwest University Chongqing 400715 P. R. China; ^2^ The State Key Laboratory Breeding Base of Basic Science of Stomatology (Hubei‐MOST) and Key Laboratory of Oral Biomedicine Ministry of Education School and Hospital of Stomatology Wuhan University Wuhan 430079 P. R. China; ^3^ Key Laboratory of Laser Technology and Optoelectronic Functional Materials of Hainan Province College of Chemistry and Chemical Engineering Hainan Normal University Haikou 571158 P. R. China

**Keywords:** cancer immunotherapy, pH cascaded responsiveness, pyroptosis, size conversion, supramolecular prodrug

## Abstract

Effective pyroptosis induction is a promising approach to potentiate cancer immunotherapy. However, the actual efficacy of the present pyroptosis inducers can be weakened by successive biological barriers. Here, a cascaded pH‐activated supramolecular nanoprodrug (PDNP) with a stepwise size shrinkage property is developed as a pyroptosis inducer to boost antitumor immune response. PDNPs comprise multiple poly(ethylene glycol) (PEG) and doxorubicin (DOX) drug–polymer hybrid repeating blocks conjugated by ultra‐pH‐sensitive benzoic imine (bzi) and hydrazone (hyd) bonds. The PEG units endow its “stealth” property and ensure sufficient tumor accumulation. A sharp switch in particle size and detachment of PEG shielding can be triggered by the acidic extracellular pH to achieve deep intratumor penetration. Following endocytosis, second‐stage size switching can be initiated by more acidic endolysosomes, and PDNPs disassociate into ultrasmall cargo to ensure accurate intracellular delivery. The cascaded pH activation of PDNPs can effectively elicit gasdermin E (GSDME)‐mediated pyroptosis to enhance the immunological response. In combination with anti‐PD‐1 antibody, PDNPs can amplify tumor suppression and extend the survival of mice, which suggests a powerful immune adjuvant and pave the way for high‐efficiency immune checkpoint blockade therapy.

## Graphical abstract

A cascaded pH‐activated supramolecular nanoprodrug (PDNP) with mutistage size shrinkage property is developed to combat the successive drug delivery barriers. The size‐transformable PDNPs can precise intracellular delivery drug for effectively eliciting pyroptosis and augmenting antitumor immune response, favorable for boosting checkpoint blockade‐based immunotherapy.

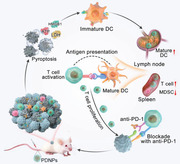

## 1 Introduction

The immune system has a high degree of plasticity, which significantly affects tumor progression.^[^ ^1^ ^]^ It is constantly regulated by immune elimination, equilibrium, and escape in the tumor microenvironment (TME).^[^ ^2^ ^]^ Checkpoint blockade‐based immunotherapy has emerged as a promising clinical approach for malignancies that can effectively alleviate immune evasion by impeding the binding of programmed death receptors and their ligands, thereby increasing the aggressiveness of the host immune system against tumor cells.^[^ ^3^ ^]^ However, its practical efficacy has long been limited by inferior activation of the immune response.^[^ ^4^ ^]^ Accumulating evidence suggests that tumors possessing high immunogenicity are pivotal for modulating the immunosuppression and boosting the immune response, which directly affects the activation and infiltration of cytotoxic T lymphocytes (CTLs).^[^ ^5^ ^]^ Recent findings on pyroptosis have elucidated its association with adaptive immunity, providing new insights for tumor immunotherapy.^[^ ^6^ ^]^ Pyroptosis is an inflammatory form of gasdermin (GSDM)‐mediated programmed cell death induced by various stimuli, such as viral, bacterial, and chemotherapy drugs.^[^ ^7^ ^]^ Cleavage of the GSDM protein family by active caspase releases the GSDM‐N fragments that punch holes in the cytomembrane, resulting in morphological variation involving membrane blebbing, cell rupture, and the leakage of cytokines, which can directly activate the immune system and positively regulate the immune response.^[^ ^8^ ^]^ A variety of chemotherapeutic drugs, such as doxorubicin (DOX), have shown to induce caspase‐3‐mediated pyroptosis in tumor cells with high gasdermin E (GSDME) expression, which potentiates the immunogenicity of tumors and elicits robust antitumor immunity.^[^ ^9^ ^]^ Despite the potential of pyroptosis for the enhancement of immunotherapy, the actual efficacy of the present pyroptosis inducers can be weakened by the successive biological barriers during drug delivery, resulting in failure to achieve the expected efficiency and causing severe adverse effects.^[^ ^10^ ^]^

Current nanodelivery systems can partially address these issues.^[^ ^11^ ^]^ For example, clinically approved PEGylated liposomal doxorubicin (Doxil) effectively enhanced tumor accumulation and relieved adverse effects.^[^ ^12^ ^]^ However, the therapeutic outcomes show no considerable improvements compared to monotherapy.^[^ ^13^ ^]^ Most nanomedicines fail to overcome the successive restrains of complicated delivery journeys, because there are different requirements for the properties of nanomedicines at different delivery phases.^[^ ^14^ ^]^ Theoretically, there is a diminishing “size ladder” principle for nanomedicines to be transported from the blood to tumor nucleus.^[^ ^15^ ^]^ Larger nanoparticles (≈100 nm) tend to accumulate at tumor sites via the enhanced permeability and retention effect, while the dense extracellular matrix, interstitial fluid pressure, and a high degree of hypovascularity hamper their deep penetration and retention in the perivascular regions of solid tumors.^[^ ^16^ ^]^ It has been reported that nanoparticles (≈30 nm) can alleviate diffusional resistance in the tumor interstitial space and facilitate intratumor penetration, whereas they are easily eliminated during blood circulation due to the poor retention effect.^[^ ^15^, ^17^ ^]^ Moreover, it requires the nanoparticles with smaller size to accurately enter the nucleus due to the nuclear pore threshold (<9 nm).^[^ ^18^ ^]^ Although most nanomedicines can surmount some of these restraints, any one of the unconquered obstacles can be an Achilles’ heel lessens the final drug amounts inside the tumor cells.

**Scheme 1:**
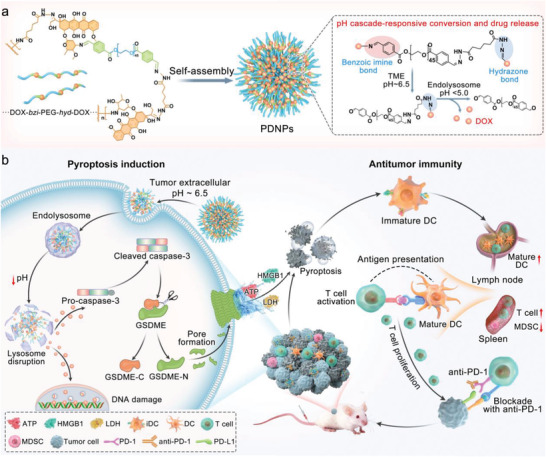
Schematic illustration of size‐transformable supramolecular nanoprodrug (PDNP)‐mediated cancer chemo‐immunotherapy. A) Chemical structure and fabrication of PDNPs and the mechanism of pH cascade‐responsive conversion and drug release. B) Illustration of PDNP‐induced pyroptosis and positive regulation of antitumor immunity.

Herein, we present a drug–polymer hybrid supramolecular nanoprodrug (PDNP) as a pyroptosis inducer to potentiate tumor immunogenicity for a robust antitumor immune response (**Scheme** **1**). PDNPs are fabricated by multiple drug–polymer repeating units, which are comprised of PEGylated DOX blocks, integrated by two different acid‐cleavable linkers to respond to the increasing acidity of the TME. At physiological pH, the PDNPs retain the “stealth” property of PEG to ensure sufficient tumor accumulation. Upon arrival at the acidic TME (pH ≈ 6.5), PDNPs begin to detach from PEG shielding and rapidly dissociate into small particles because the protonation of the ultra‐pH‐sensitive benzoic imine bond for deep tumor penetration. After endocytosis, second‐stage size shrinkage occurs because the cleavage of the hydrazone bond in response to the more acidic endolysosome (pH ≈ 5.0), resulting in complete decomposition of PDNPs and release of DOX into the nucleus. Effective penetration and accurate release of drugs in the tumor can effectively provoke GSDME‐mediated pyroptosis, which facilitates dendritic cell (DC) maturation, prime CTL proliferation, and inhibiting myeloid‐derived suppressor cells (MDSCs), thereby boosting the antitumor immune response. Owing to their pyroptosis‐induced immune‐potentiating effects, PDNPs can sensitize tumors to anti‐PD‐1 checkpoint blockade therapy, amplify therapeutic efficacy, and prolong survival; in cooperation with immune checkpoint inhibitors, they may be a powerful clinical candidate for boosting cancer chemo‐immunotherapy.

## 2 Results and Discussion

**Figure 1:**
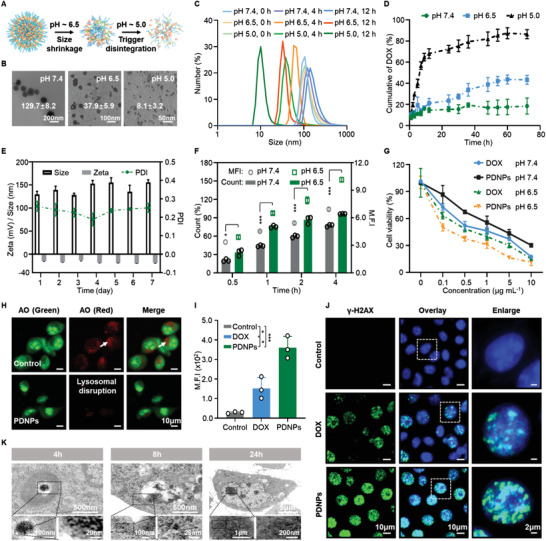
Characterization and working mechanism of PDNPs. A) Schematic illustration of pH‐triggered sequential conversion of PDNPs. B) TEM images of PDNPs at pH 7.4, 6.5, and 5.0. C) Particle size distribution of PDNPs at different pH values over time. D) The drug release profiles of PDNPs in PBS solutions with different pH values (data presented as mean ± SD, *n* = 3). E) The stability of PDNPs within 7 days (data presented as mean ± SD, *n* = 3). F) Cellular uptake count (histogram) and the mean fluorescence intensity (M.F.I) (scattergram) of PDNPs in CT26 cells at pH 7.4 or 6.5 (data presented as mean ± SD, *n* = 3, *P*‐values are calculated using Student's *t*‐test, *P < 0.05, ***P < 0.001). G) Cell viability at pH 7.4 or 6.5 (data presented as mean ± SD, *n* = 3). H) Acridine orange (AO) staining to indicate lysosomal disruption in CT26 cells treated with PDNPs. I,J) The M.F.I and CLSM images of DNA damage in CT26 cells incubated with PDNPs and free DOX (data presented as mean ± SD, *n* = 3, *P*‐values are calculated using one‐way ANOVA, *P < 0.05, ***P < 0.001). K) TEM images of CT26 cell treated with PDNPs at different time points.

PDNPs were synthesized by a two‐kind aminaldehyde reaction of aldehylated PEG blocks and hydrazone bond‐modified DOX prodrugs (Figure S1, Supporting Information), forming a drug–polymer hybrid supramolecular prodrug (DOX‐*bzi*‐PEG‐*hyd*‐DOX). The chemical structures were confirmed using ^1^H NMR spectroscopy and an FTIR spectrophotometer (Figures S2–S5, Supporting Information). The characteristic peak of DOX in the fluorescence spectrum and ultraviolet (UV) absorption was observed in the PDNPs, confirming the successful integration of DOX (Figure S6, Supporting Information). The number‐average molecular weight (M_n_) and weight‐average molecular weight (M_w_) of PDNPs were 1.2 × 10^4^ and 2.6 × 10^4^, respectively, demonstrating that PDNPs were integrated by multiple repeated drug–polymer blocks (Figure S7, Supporting Information). The unique structure of PDNPs endows it with a high drug loading capacity of 12.6 ± 0.7 wt%, as determined by UV–vis absorbance (Figure S8, Supporting Information). The PDNPs showed a negative surface charge of −14.6 ± 1.4 mV (Figure S9, Supporting Information). The hemolysis rate of PDNPs was lower than 5% in the range of 1–200 µg mL^−1^, indicating their superior blood compatibility (Figure S10, Supporting Information). The stability of PDNPs was examined by dynamic light scattering (DLS) measurements over a period of 7 days in PBS, and there were slight but negligible changes in size and zeta potential (**Figure** **1**E). Furthermore, PDNPs maintained good stability and polydispersity in DMEM or PBS with 10% FBS at 48 h (Figure S11, Supporting Information), suggesting that PDNPs possess desirable stability during blood circulation, thus ensuring effective tumor accumulation.

To test cascaded acid sensitivity, PDNPs were incubated in solutions with different pH values. The mechanism of pH‐triggered sequential conversion is illustrated in Figure 1A, and its morphology and size were measured using transmission electron microscopy (TEM) and DLS. At pH 7.4, the PDNPs showed a spherical shape with an average size of 129.7 ± 8.2 nm. PDNPs shrunk to 37.9 ± 5.9 nm at pH 6.5 and finally completely decomposed into 8.1 ± 3.2 nm at pH 5.0 (Figure 1B), which was consistent with the DLS results (Figure S12, Supporting Information). The variations in the particle size of PDNPs incubated in different pH solutions over time are shown in Figure 1C, confirming the stepwise size shrinkage property of PDNPs owing to the cleavage of acid‐labile linkage and PEG detachment.

To investigate the pH‐triggered drug release behavior of PDNPs, cumulative DOX release from PDNPs was evaluated in PBS at pH 7.4, 6.5, and 5.0. The release profile of the PDNPs showed a sustained release of DOX over 72 h under diverse conditions. There was a rapid drug release of 43.4% and 86.6% at pH 6.5 and 5.0, respectively; however, only 18.6% DOX was released from PDNPs at pH 7.4 (Figure 1D). PDNPs showed an increase in cumulative drug release at pH 6.5 compared with that at pH 7.4, which was ascribed to the protonation of the benzoic imine moiety in PDNPs in a weakly acidic environment. The drug release rate of PDNPs at pH 5.0 was faster than that at pH 6.5 because of the cleavage of the hydrazone bond at more acidic pH conditions. These results were consistent with the fluorescence spectrometer, which showed a significant increase in fluorescence intensity of ≈2.2‐fold and ≈2.8‐fold at pH 6.5 and 5.0, respectively, compared to that at pH 7.4, confirming that the sequential dissociation of PDNPs was triggered at different pH ranges (Figure S13, Supporting Information).

Although PEGylation has been proven to be an effective strategy for prolonging circulation, the resulting inefficiency of cellular uptake remains a concern.^[^ ^19^ ^]^ By virtue of the introduction of an acid‐labile linkage, dePEGylation can be triggered in response to an acidic TME, which is capable of amplifying the intracellular delivery efficiency by PEG deshielding. Therefore, we investigated the acid‐dependent intracellular activation of PDNPs in murine colon cancer (CT26) cell lines. A strong fluorescence signal of DOX was observed in CT26 cells after 4 h of coincubation with PDNPs at pH 7.4, while there was an obvious signal augmentation with the pH value decreasing to 6.5 (Figure S14, Supporting Information). Flow cytometry was used to further quantify the amount of DOX internalized by the PDNPs (Figure S15, Supporting Information). At pH 6.5, the percentage of positive cells incubated with PDNPs after 4 h was ≈1.2‐fold higher than that at pH 7.4. The mean fluorescence intensity of the positive cells also exhibited a significant elevation at pH 6.5 (Figure 1F), confirming the enhancement of cellular uptake attribute to acid‐triggered PEG detachment. The cytotoxicity of PDNPs was evaluated by MTT and live‐staining assays in CT26 cell lines (Figure 1G; Figure S16, Supporting Information). PDNPs exhibited dose‐dependent antitumor efficacy, and cell viability was dramatically reduced upon incubation with PDNPs at pH 6.5 than that at pH 7.4. Moreover, the half‐inhibitory concentration (IC_50_) value of the PDNPs in CT26 cells at pH 7.4 was 6.8 μg/mL, whereas it was 3.2 μg/mL at pH 6.5 (Figure S17, Supporting Information). The increased cytotoxicity of PDNPs at pH 6.5 could be ascribed to more efficient cellular uptake and higher DOX release under acidic conditions.

To explore the intracellular trafficking pathway and working mechanism of PDNPs, we first evaluated the colocalization efficiency of PDNPs with lysosomes. As shown in Figure S18 of the Supporting Information, the overlapping fluorescence signal between PDNPs and endolysosomes first increased and then decreased over time, indicating that PDNPs could effectively target and escape from lysosomes. The Pearson correlation coefficient quantitatively affirmed the time‐determined escape of PDNPs from lysosomes, which was ascribed to the ionization of PDNPs in the acidic compartment, followed by the “pH sponge effect” giving rise to lysosomal damage. We then evaluated the disruption of lysosomes by PDNPs using acridine orange staining. As shown in Figure 1H, the red signals assigned to lysosomes almost disappeared after 12 h of incubation with PDNPs, and the green fluorescence reflected a shrinking morphology due to cell apoptosis. As a genotoxic therapeutic agent, DOX induces apoptosis by interfering with topoisomerase II‐mediated DNA replication and transcription. Thus, we detected the degree of DNA damage caused by the PDNPs using *γ*‐H2AX antibody immunofluorescence staining (Figure 1I,J). Vast accumulation of green fluorescence was observed in the nucleus after PDNPs treatment for 24 h, indicating that PDNPs could elicit intense DNA damage. Furthermore, we observed the intracellular trafficking pathway of PDNPs in CT26 cells using TEM. As shown in Figure 1K, a mass of small nanoparticles with a size of ≈10 nm rapidly accumulated in lysosomes after 4 h of incubation, confirming the endocytosis mechanism of PDNPs in acidic endolysosomes, with a size‐transformable performance. After 8 h, the lysosomes were disrupted and the nanoparticles were prone to escape from them into the cytoplasm. After incubation for 24 h, many tiny particles covered the entire cytoplasm and tended to accumulate in the nucleus, leading to apoptosis. Taken together, these results implied that PDNPs could dissociate into small nanoparticles in response to the acidic endolysosomes and escape from the endolysosomal chamber due to the proton sponge effect, avoiding elimination by the exocytosis pathway without drug release, facilitating active drug molecule access to the nucleus, and inducing cell apoptosis.

**Figure 2:**
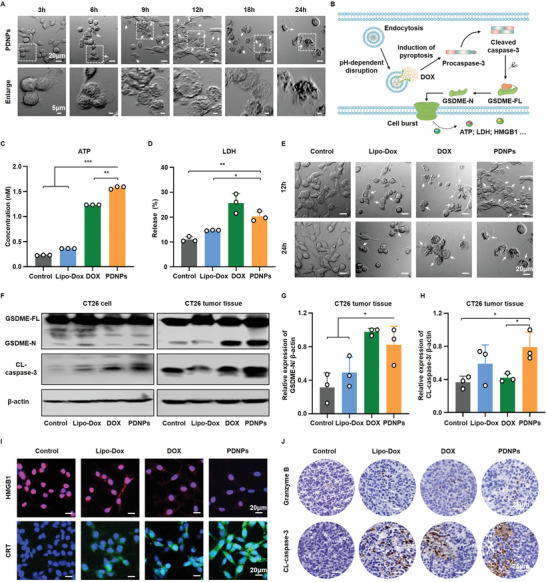
Pyroptosis and immunostimulatory behaviors of PDNPs. A) Representative bright‐field CLSM images of CT26 cells treated with PDNPs at different time period. Arrows denote the pyroptotic cells. B) Schematic illustration of PDNPs‐induced pyroptosis mechanism. C,D) The release of ATP and LDH from CT26 cells after different treatments (Data presented as mean ± SD, *n* = 3, *P*‐values are calculated using one‐way ANOVA, *P < 0.05, **P < 0.01, ***P < 0.001). E) Representative bright‐field CLSM images of CT26 cells with different treatments. F) Western blotting detection of full‐length GSDME (GSDME‐FL), GSDME‐N terminal, and Cleaved caspase‐3 (CL‐caspase‐3) in CT26 cells and tumor tissues upon PDNPs treatment (data presented as mean ± SD, *n* = 3). G,H) The relative GSDME‐N expression levels and CL‐caspase‐3 expression levels in CT26 tumor tissues (data presented as mean ± SD, *n* = 3, *P*‐values are calculated using one‐way ANOVA with Bonferroni correction, *P < 0.05). I) The representative CLSM images of HMGB1 and CRT in CT26 cells. J) The representative immunohistochemical staining of granzyme B and CL‐caspase‐3 in tumor tissues upon different treatments.

Effective antigen presentation is an indispensable prerequisite for T cell activation. Recent studies have asserted that DOX induces pyroptosis by activating caspase‐3 to cleave GSDME.^[^ ^9a^ ^]^ The resulting N‐terminal domains of GSDME would elicit membrane perforation and cell burst, accompanied by secretion of high‐mobility group box 1 (HMGB1) and release of lactate dehydrogenase (LDH) and adenosine triphosphate (ATP) to promote DCs maturation and antigen presentation to enhance the immunological response (Figure **2**B). Thus, we speculated that PDNPs could effectively induce pyroptosis to stimulate the immune response. First, we examined whether CT26 cells exhibit the morphological characteristics of pyroptosis upon PDNPs treatment. As shown in Figure 2A, features of pyroptosis with cell swelling and ballooning from the plasma membrane were observed after 6 h of treatment with PDNPs. At 12 h, pyroptosis occurred in most cells and the cells tended to burst after 24 h of treatment with PDNPs. Considering that PEGylated liposomal doxorubicin (Lipo‐Dox) with a particle size of ≈180 nm is an FDA approved clinical preparation, we used it for comparison (Figure S19, Supporting Information). As shown in Figure 2E, the images of cells with different treatments displayed an increased number of pyroptotic cells. Next, we determined the release of ATP and LDH in CT26 cell line, and the high release content of ATP and LDH was measured in the supernatant of PDNP‐treated cells (Figure 2C,D).

We next detected pyroptosis‐related protein expression in vitro and in vivo using western blotting. As shown in Figure 2F–H and Figure S20 (Supporting Information), high‐level expression of GSDME‐N and cleaved caspase‐3 (CL‐caspase‐3) was observed in PDNP‐treated CT26 cell lines and tumor tissues, confirming that active caspase‐3 effectively cleaved the linkage of GSDME to generate the GSDME‐N domain and induce pyroptosis. Similar results were observed in murine breast cancer cell lines (4T1) (Figure S21, Supporting Information). Thereafter, cell membrane exposure of calreticulin (CRT) and extracellular release of HMGB1 were evaluated to verify the enhancement of PDNP‐induced immunogenicity. Figure 2I shows obvious HMGB1 release and CRT exposure in PDNP‐treated cells. Quantitative analysis showed that PDNPs significantly elevated HMGB1 release and CRT exposure, which were ≈2.7‐ and ≈6.4‐fold higher than those in the control group (Figure S22, Supporting Information). In addition, granzyme B can directly shear GSDME and activate pyroptosis in tumor cells. Thus, we examined the expression of granzyme B and CL‐caspase‐3 in the tumor tissues by immunohistochemistry, and the local increase was clearly observed in the PDNP‐treated group (Figure 2J; Figure S23, Supporting Information).

**Figure 3:**
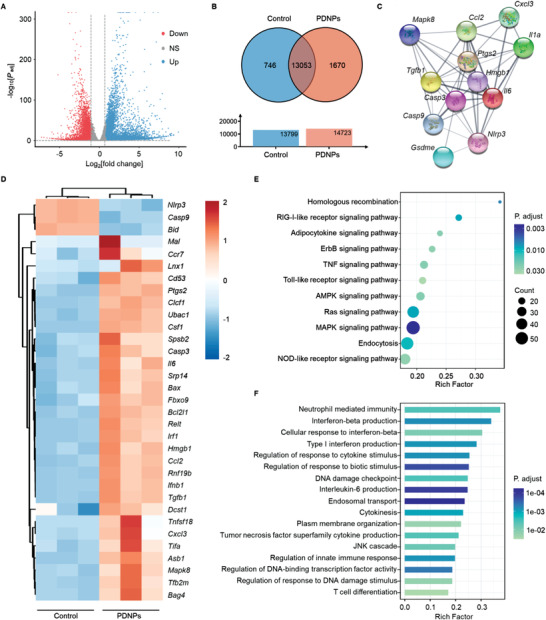
RNA sequencing analysis. A) Volcano plot of the distributions of differentially expressed genes after PDNPs treatment (|log_2_ fold change| > 1, adjusted *P‐*value < 0.05). B) Venn diagram of all expressed genes in each group. C) Functional associated networks of the differentially expressed genes from the STRING database. D) Heatmap analysis of mRNA expression levels of genes involved in pyroptosis and immune system. E) KEGG pathway analysis and F) GO enrichment after PDNPs treatment. Data presented as mean ± SD, *n* = 3.

To further understand the mechanisms of pyroptosis and related immune responses induced by PDNPs, we evaluated gene expression by RNA‐seq in CT26 cells after treatments, and the principal component analysis, as shown in Figure S24 of the Supporting Information. As depicted in the volcano plot (**Figure** **3**A), 6941 differential genes were detected in the PDNP‐treated groups compared with the control group (|log_2_ fold change| > 1 and adjusted *P*‐value < 0.05), including 3142 upregulated genes and 3799 downregulated genes. The Venn diagram shows all the expressed genes in the control and PDNPs groups (Figure 3B). We then analyzed the mRNA expression levels of genes associated with pyroptosis and the immune system and found that genes related to inflammatory cytokines, antigen processing and presentation, and T cell functional molecules were distinctly upregulated in the PDNP‐treated group (Figure 3D). As shown in Figure 3C, these upregulated genes were closely associated with each other and participated individually or jointly in the innate and adaptive immune responses. Kyoto Encyclopedia of Genes and Genomes (KEGG) pathway enrichment analysis indicated that after PDNPs treatment, differentially expressed genes were enriched in inflammatory‐related signaling pathways, apoptosis‐mediated signaling pathways, and immune response‐associated signaling pathways, such as TNF signaling pathways, NOD‐like receptor signaling pathway, MAPK signaling pathway, RIG‐I‐like receptor signaling pathway, and Toll‐like receptor signaling pathway (Figure 3E). Gene ontology (GO) enrichment analysis further revealed a greater rich ratio upon treatment with PDNPs being mainly involved in inflammatory responses, regulation of response to cytokine stimulus, innate immune response, and T cell differentiation (Figure 3F). These results confirm that PDNPs treatment efficiently elicit pyroptosis to potentiate tumor immunogenicity for a robust antitumor immune response.

**Figure 4:**
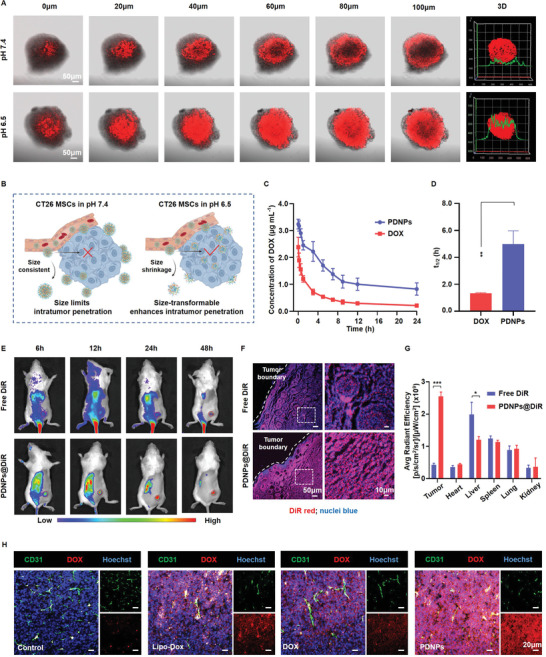
Penetration, pharmacokinetics, and biodistribution studies of PDNPs. A) The representative CLSM images of CT26 MCSs at varied depths upon incubation with PDNPs for 6 h at pH 7.4 or 6.5 (data presented as mean ± SD, *n* = 3). B) Schematic illustration of the acid‐triggered size‐transformable PDNPs for enhanced intratumor penetration in CT26 MCSs. C) Plasma concentration of DOX versus time of PDNPs upon administration of PDNPs and free DOX in S.D. rats (data presented as mean ± SD, *n* = 3). D) The circulating half‐life (*t*_1/2_) of PDNPs and free DOX in S.D. rats (data presented as mean ± SD, *n* = 3, *P*‐values are calculated using Student's *t*‐test, **P < 0.01). E) The representative in vivo NIR imaging of free DiR and PDNPs@DiR in CT26‐bearing mice. Red circles denote the tumor region (data presented as mean ± SD, *n* = 3). F) The representative CLSM images of the distribution and penetration of PDNPs in the slices of CT26 tumor tissue. G) Quantitative analysis of the excised tumors and organs at 48 h postinjection (data presented as mean ± SD, *n* = 3, *P*‐values are calculated using Student's *t*‐test, ***P < 0.001). H) The representative immunofluorescence staining of excised tumor tissues after treatments for penetration studies. (DAPI: blue; DOX: red; blood vessels: green, stained with CD31 antibody.)

The potency of sufficient tumor accumulation and deep intratumor penetration seems contradictory for nanomedicine. Nanoparticles with a small size have been proven to be favorable for diffusion into tumors, but are easily cleared by the blood circulation system. Since PDNPs possess size‐transformable properties in response to the acidic tumor extracellular pH, they were expected to show enhanced intratumor infiltration and simultaneously retain the capacity of long blood circulation. To confirm this, 3D tumor models fabricated by CT26 multicellular spheroids (MCSs) were utilized to evaluate the penetration capability of PDNPs in vitro and the schematic illustration is shown in **Figure** **4**B. After incubation with PDNPs in pH 7.4 and 6.5 medium for 6 h, the red fluorescence of PDNPs perfused throughout the MCSs at pH 6.5, in contrast with the limited distribution around the MCSs periphery at pH 7.4 (Figure 4A; Figure S25, Supporting Information), demonstrating that the acid‐triggered size conversion was a feasible strategy to facilitate particle traversing of the dense tumor matrix. The pharmacokinetics of PDNPs were then studied by detecting the plasma levels of DOX after intravenous injection in S.D. rats. The circulating half‐life (*t*_1/2_) and AUC value of PDNPs were ≈3.7‐fold and ≈2.9‐fold higher than those of free DOX, respectively (Figure 4C,D; Figure S26, Supporting Information), and the pharmacokinetics parameters were presented in Table S1 of the Supporting Information, indicating the preferable retention time of PDNPs in vivo.

Thereafter, the in vivo tumor accumulation and distribution of PDNPs were investigated after their administration into CT26 tumor‐bearing mice. We first loaded the lipophilic near‐infrared (NIR) fluorescent dye DiR into the PDNPs (denoted PDNPs@DiR). The in vivo NIR fluorescence images showed that the PDNPs@DiR‐treated group showed more intense fluorescence than those of free DiR treated group at 6, 12, 24, and 48 h postinjection in the tumor region (Figure 4E; Figures S27 and S28, Supporting Information). After 48 h, the tumor fluorescence in the PDNPs@DiR treated group was still strong, and the fluorescence intensity was ≈6.1‐fold higher than that of the free DiR‐treated group (Figure 4G). We then examined the tumor sections using CLSM. As shown in Figure 4F, extensive signals from the PDNPs@DiR group were observed in the tumor parenchyma. By contrast, the signals of the free DiR group were confined to the peripheral tumor, and it was difficult to infiltrate deeply into the tumors. The corresponding quantitative analysis of the DiR distribution is shown in Figure S29 of the Supporting Information. We further investigated whether PDNPs could effectively extravasate from tumor vessels by examining intratumor distribution with immunofluorescence staining. After in vivo experiments, the harvested tumors were stained with anti‐CD31 antibodies that indicated tumor vascular endothelium. As shown in Figure 4H and Figure S30 (Supporting Information), PDNPs could highly diffuse from the tumor vessels and have a more extensive fluorescence distribution. By contrast, the signals of Lipo‐Dox were almost confined to the perivascular area of the tumor, and it was difficult to penetrate into the deep regions, which might be ascribed to the limitation of particle size. These results indicate that size‐transformable PDNPs could address the need for both long blood circulation and deep intratumor penetration.

**Figure 5:**
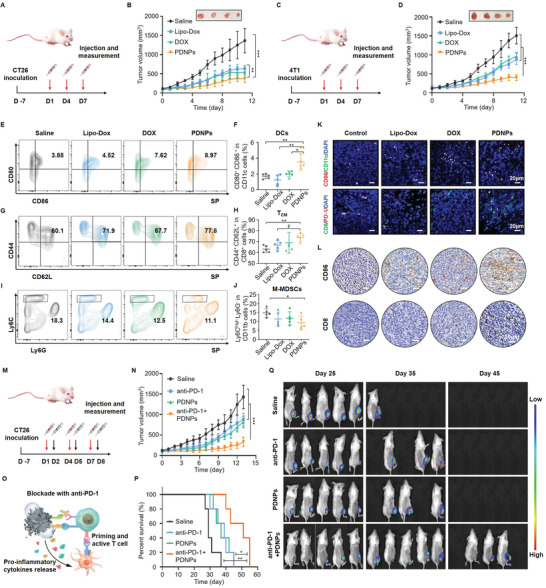
In vivo antitumor activity and enhanced immune responses for combined anti‐PD‐1 therapy. A–D) Therapeutic schedule for tumor treatments in CT26 or 4T1‐bearing mice models and the corresponding growth curves upon different treatments. E,F) Flow cytometric analysis and quantitation of CD80^+^ CD86^+^ DCs on CD11c cells in spleen (SP). G,H) Flow cytometric analysis and quantitation of CD44^+^ CD62L^+^ central memory T (T_CM_) cells on CD8^+^ cells in SP. I,J) Flow cytometric analyses and quantitation of Ly6C^high^ Ly6G^−^ M‐MDSCs on CD11b cells in SP. K,L) The representative immunofluorescence and immunohistochemical analysis of DC maturation and T cell infiltration in the tumor. M) Therapeutic strategy of PDNPs combined with anti‐PD‐1 therapy in CT26‐bearing mice (red arrows denote PDNPs administration; black arrows denote anti‐PD‐1 administration). N) Growth curves of the combination therapy upon different treatments in CT26‐bearing mice. O) Schematic illustration of the mechanism of PDNPs‐induced pyroptosis for effective anti‐PD‐1 checkpoint blockade therapy. P) Survival curves of the combination therapy upon different treatments. Q) Bioluminescence imaging of CT26‐bearing mice during the experiment. Data presented as mean ± SD, *n* = 5, *P*‐values are calculated using one‐way ANOVA, *P < 0.05, **P < 0.01, ***P < 0.001.

Next, the in vivo antitumor efficiency of PDNPs was evaluated in BALB/c mice bearing CT26 or 4T1 tumor xenografts (**Figure** **5**A–D). All animal experiments were conducted in accordance with the animal research guidelines approved by the Experimental Animal Ethics Committee of the School and Hospital of Stomatology, Wuhan University (S07920080J). Tumor volume increased rapidly in the saline‐treated group. Lipo‐Dox and free DOX treatments resulted in moderate therapeutic effects, with ≈43% and ≈49% (CT26 tumor model) or ≈37% and ≈35% (4T1 tumor model) inhibition rates, respectively, versus saline treatment. By contrast, PDNP treatments resulted in the highest antitumor activity, with ≈74% (CT26 tumor model) or ≈85% (4T1 tumor model) tumor suppression (Figures S31 and S32, Supporting Information). Hematoxylin and eosin staining and TUNEL staining in both tumor models showed evident tumor necrosis and increased cell apoptosis induced by PDNPs. Ki67 staining revealed the lowest level of proliferating cells after treatment with PDNPs (Figures S33 and S34, Supporting Information). Furthermore, no significant weight loss, spleen inflammation, or cardiac tissue damage were observed after PDNPs treatment (Figures S35–S40, Supporting Information). Hematological assays showed no obvious abnormalities, indicating that the PDNPs were of prominent biosafety (Figure S41, Supporting Information). These results demonstrate that the size‐transformable PDNPs have prolonged circulation time, enhanced tumor accumulation, deep intratumor penetration, effective cellular uptake, and sufficient drug release, thereby resulting in superior therapeutic outcomes and reduced adverse effects.

Having demonstrated the immune‐potentiating effects of PDNPs by inducing pyroptosis, we further explored whether PDNPs could evoke immune responses by harvesting spleens and tumors from CT26 tumor‐bearing mice after treatment using flow cytometry, immunofluorescence, and immunohistochemical analysis. The gating strategies used in flow cytometry analysis are shown in Figures S42–S44 of the Supporting Information. The results revealed that the percentage of mature CD80^+^ CD86^+^ DCs and CD44^+^ CD62L^+^ central memory T cells (T_CM_) within the spleen were obviously upregulated after PDNPs treatment for immune response (Figure 5E–H). Furthermore, MDSCs in the TME are known to be the main obstacle of antigen presentation and CTLs function. Remarkably, a decrease in the percentage of M‐MDSCs (Ly6C^high^ Ly6G^−^) was observed within the spleen after PDNPs treatment, indicating that PDNPs could effectively reduce M‐MDSCs and thus ameliorate the immunosuppressive microenvironment (Figure 5I,J, Supporting Information). Moreover, this enhanced immunity was further confirmed in tumors by immunofluorescence and immunohistochemical staining. As shown in Figure 5K and Figure S45 (Supporting Information), the coexpression of CD86^+^ CD11c^+^, CD11c^+^ CD103^+^, and CD8^+^ CD103^+^ cell populations in tumor tissues exhibited a striking increase in the PDNPs group, indicating that PDNPs could effectively promote DCs maturation and T cell infiltration. The expression of CD8^+^ PD‐1^+^ T cells in tumors verified the effect of PDNPs on the enhancement of CTLs and the relief of CD8^+^ T cells exhaustion. In addition, immunohistochemical staining and the corresponding quantitative analysis of CD86, CD8, CD103, CD11c, and MDSCs marker Arginase‐1 further confirmed the enhanced immunogenicity induced by PDNPs (Figure 5L; Figures S46 and S47, Supporting Information).

Inspired by the results that PDNPs could effectively induce pyroptosis to boost antitumor immunity, we next explored whether the combination therapy of PDNPs plus the checkpoint inhibitor anti‐PD‐1 could amplify antitumor efficacy and extend survival through enhanced immune responses. As depicted in the treatment schedule, PDNPs were administrated preferentially to rejuvenate CD8^+^ T cells, and anti‐PD‐1 was injected intraperitoneally the next day to block PD‐1 in BALB/c mice bearing CT26 or 4T1 tumor xenografts (Figure 5M; Figure S51A, Supporting Information). As expected, the combination of PDNPs and anti‐PD‐1 sharply enhanced the therapeutic efficiency compared with the single therapy modality (Figure 5N; Figures S48, S49, and S51B–D, Supporting Information), which might be ascribed to the immune‐potentiating effects of PDNPs that reinforce T cell responses. In addition, tumor growth was monitored using bright field and IVIS Lumina imaging, suggesting that the combination therapy dramatically extended survival compared to individual treatment alone (Figure 5P,Q; Figure S50, S51E, Supporting Information). Collectively, PDNPs are capable of sensitizing tumors to anti‐PD‐1‐mediated immune checkpoint blockade therapy by enhancing antitumor immune response, thus exerting amplified therapeutic efficacy and extended survival.

## 3 Conclusions

In summary, we have successfully fabricated a simple, tuneable, and safe supramolecular nanomedicine with multistage pH‐triggered size shrinkage and dePEGylation to combat cascaded drug delivery barriers and precisely delivery DOX to effectively elicit pyroptosis and augment antitumor immune response. The well‐tailored PDNPs exhibited high stability and stealth in blood circulation to avert nonspecific interactions with plasma proteins and clearance of the mononuclear phagocyte system, resulting in favorable pharmacokinetics and high tumor accumulation. A sharp change in particle size and detachment of PEG shielding in response to the acidic extracellular pH remarkably promoted extravasation from the tumor vasculature and infiltration into tumors. Subsequently, the second‐stage size shrinkage was initiated by lower acidic endolysosomes, and the ultrasmall cargo size ensured successful entry into the nucleus for the precise delivery of DOX. The cascaded pH activation of PDNPs effectively elicited GSDME‐mediated pyroptosis and potentiated immunogenicity, thus facilitating DC maturation and CD8^+^ T cell infiltration, priming CTL proliferation, and boosting the antitumor immune cycle. In combination with anti‐PD‐1, PDNPs achieved amplified tumor suppression and significantly extended survival. PDNPs that elicit vigorous pyroptosis may be powerful yet promising immune adjuvants to augment checkpoint blockade‐based immunotherapy efficacy via generating a robust immune response.

## Conflict of Interest

The authors declare no conflict of interest.

## Supporting information

Supporting Information

## Data Availability

The data that support the findings of this study are available from the corresponding author upon reasonable request.
