# Supplementary material for: Stepwise Size Shrinkage Cascade‐Activated Supramolecular Prodrug Boosts Antitumor Immunity by Eliciting Pyroptosis
Source: Adv Sci (Weinh). 2022 Jul 22;9(26):2203353. doi: 10.1002/advs.202203353 (PMC9475545; doi:10.1002/advs.202203353)
Supplement: Supplementary file 1 — Supporting Information [file ADVS-9-2203353-s001.pdf]

ADVANCED SCIENCE
Open Access

Supporting Information

for *Adv. Sci.*, DOI 10.1002/advs.202203353

Stepwise Size Shrinkage Cascade-Activated Supramolecular Prodrug Boosts Antitumor Immunity by Eliciting Pyroptosis

Meng-Yun Liang, Meng-Jie Zhang, Wei Qiu, Yao Xiao, Meng-Jie Ye, Peng Xue, Yue-Jun Kang, Zhi-Jun Sun and Zhigang Xu**

Supporting Information

Stepwise Size Shrinkage Cascade-Activated Supramolecular Prodrug Boosts Anti-Tumour Immunity by Eliciting Pyroptosis

Meng-Yun Liang, Meng-Jie Zhang, Wei Qiu, Yao Xiao, Meng-Jie Ye, Peng Xue, Yue-Jun Kang, Zhi-Jun Sun* and Zhigang Xu*

#M.-Y. Liang and M.-J. Zhang contribute equally to this work

M.-Y. Liang, W. Qiu, M.-J. Ye, P. Xue, Y.-J. Kang, Prof. Z. G. Xu
State Key Laboratory of Silkworm Genome Biology, School of Materials and Energy & Chongqing Key Laboratory of Soft-Matter Material Chemistry and Function Manufacturing
Southwest University,
Chongqing 400715, P. R. China
E-mail: zgxu@swu.edu.cn (Z.G.X.).

M.-J. Zhang, Y. Xiao, Prof. Z.-J. Sun
The State Key Laboratory Breeding Base of Basic Science of Stomatology (Hubei-MOST) & Key Laboratory of Oral Biomedicine, Ministry of Education, School & Hospital of Stomatology
Wuhan University,
Wuhan 430079, P.R. China
E-mail: sunzj@whu.edu.cn (Z.J.S.).

Prof. Z. G. Xu
Key Laboratory of Laser Technology and Optoelectronic Functional Materials of Hainan Province, College of Chemistry and Chemical Engineering
Hainan Normal University,
Haikou 571158, P. R. China

Experimental Section

Materials, cell lines and animals: All chemical regents were supplied from Adamas-beta (China) and Sigma-Aldrich (USA) Co. Ltd. (China). All biological reagents were purchased from Life Technologies (China) and Beyotime Biotechnology (Shang Hai, China). The pyroptosis markers detected by western blot were as follows: GSDME (ab215191, Abcam), Cleaved caspase 3 (5A1E, Cell Signaling Technology) and anti-β-actin antibody (ab8226, Abcam). The Flow cytometry antibodies including anti-CD45-PC5.5 (30-F11, Invitrogen), anti-CD11c-FITC (N418, Invitrogen), anti-MHC II-PC7 (M5/114.15.2, Biolegend), anti-CD80-PE (16-10A1, Invitrogen), anti-CD86-APC (GL1, Invitrogen), anti-CD3-FITC (17A2, Biolegend), anti-CD4-ef450 (RM4-5, Invitrogen), anti-CD8-PC5.5 (53-6.7, Biolegend), anti-CD44-PE (IM7, Invitrogen), anti-CD62L-APC (MEL-14, eBioscience), anti-CD11b-FITC (M1/70, Biolegend) anti-Ly6C-APC (HK4, Invitrogen), anti-Ly6G-PE (1A8, Biolegend) were used. The antibodies used for immunofluorescence and immunohistochemistry were as follows: CD86 (E5W6H, CST), CD11c (D1V9Y, CST), CD8 (D4W2Z, CST), PD-1 (D7D5W, CST), CD103 (ab254182, Abcam), Arginase-1 (D4E3M, CST), Granzyme B (D6E9W, CST) and Cleaved caspase-3 (Asp175, CST). Fluorescent secondary antibodies were purchased from Abbkine (China). The *in vivo* anti-PD-1 mAb (CD279) was obtained from BioXcell (clone RMP1-14, Cat BE0146).

Murine colon carcinoma cells (CT26) and murine breast cancer cells (4T1) were obtained from American Type Culture Collection (ATCC). Both cell lines were cultured in DMEM cell culture medium, containing 10% fetal bovine serum (FBS) and 1% penicillin-streptomycin solution.

Female BABL/c mice (5-6 weeks, 18-20 g) and Sprague-Dawley (S.D.) rats (180-220 g) were all purchased from Byrness Weil biotech Ltd (Chongqing, China). All animal experiments were conducted in accordance with the animal research guidelines approved by

the Experimental Animal Ethics Committee of the School and Hospital of Stomatology, Wuhan University (S07920080J).

Synthesis of Aldehylated PEG chain (CHO-PEG-CHO): The synthesis of aldehylated PEG chain was according to the reported method with slightly modification.[1] PEG_{2000} (4.0 g, 2.0 mmol), 4-carboxybenzaldehyde (0.91 g, 6.0 mmol) and 4-dimethylamiopryidine (DMAP, 0.1 g, 0.8 mmol) were dissolved in 60 mL anhydrous dichloromethane (DCM). Subsequently, N,N'-dicyclohexylcarbodiimide (DCC, 1.86 g, 6.0 mmol) was added dropwise under ice bath and the mixture was stirred for 48 h at 25°C. After the reaction, the solution was filtered and concentrated by rotary evaporator. The resulting product was collected by precipitation with excess diethyl ether three times and dried under vacuum overnight with about 78% yield. The final product was characterized by ^{1}H NMR spectroscopy (Bruker Avance-600) in DMSO-d_6 and Fourier transform infrared (FTIR) spectroscopy (Thermo Nicolet 6700), as shown in Figure S2 and S5.

Synthesis of hydrazone bond-modified doxorubicin prodrug (DA): DOX (464 mg, 0.8 mmol) and adipic acid dihydrazide (ADH, 696.8 mg, 4.0 mmol) were dissolved in 100 mL anhydrous methanol and stirred for 30 min at 25°C under argon protection. Then 800 μL trifluoroacetic acid (TFA) was added and the reaction was maintained at 50°C overnight. After the reaction, the methanol was removed by rotary evaporation. Then the crude product was re-dissolved in ethanol and centrifuged to remove solid. The purification process was repeated twice. The solution was concentrated in vacuum and precipitated with excess diethyl ether to obtain a dark-red product (Yield% ≈ 72%). ^{1}H NMR (600 MHz, DMSO-d_6) and FTIR are shown in Figure S3 and S5.

Synthesis of drug-polymer hybrid supramolecular prodrug: CHO-PEG-CHO (600 mg) and DA (195 mg) were dissolved in 2 mL anhydrous dimethyformamide (DMF). Then 200 μL triethylamine (TEA) was added and the mixture was stirred for 24 h at 50°C. After the reaction, the solution was dialyzed (MWCO, 7.0 kDa) against methanol to remove unreacted

substance. Finally, the resulting solution was precipitated into excess diethyl ether and washed three times. The product was obtained after vacuum drying (Yield% ≈ 59%). ^{1}H NMR (600 MHz, DMSO-d_6) and FTIR are shown in Figure S4 and S5. GPC date (Figure S7) was utilized to measure the molecular weight of the drug-polymer prodrug.

Preparation and characterization of PDNPs: PDNPs were facilely prepared by nanoprecipitation method. Briefly, the supramolecular prodrug was dissolved in dimethyl sulfoxide (DMSO) based on appropriate ratio and added dropwise to Milli-Q water under ultrasonication. Afterwards, the organic solvent was removed by dialyzing (MWCO, 3.5 kDa) against Milli-Q water to obtain PDNPs.

The zeta potential and size distribution of all prepared nanoparticles at different pH values (pH 7.4, pH 6.5 or pH 5.0) were measured by dynamic light scattering (DLS, Nano ZS90, Malvern). The morphologies of the PDNPs were characterized by transmission electron microscopy (TEM, HT7800, 90 kV). Fluorescence intensity of DOX was measured by a fluorescence spectroscopy (Ex: 488 nm, Em: 560-640 nm, Shimadzu RF-5301PC, Japan). The Ultraviolet-visible (UV-vis) absorption of the PDNPs was recorded with a spectrophotometer (Shimadzu UV-1800, Japan).

Preparation of PEGylated liposomal doxorubicin (Lipo-Dox): The neutral PEGylated liposomes were prepared using the film hydration method. Briefly, the lipid mixtures consisted of DSPE-PEG$_{2000}$: Cholesterol (CHOL): Hydrogenated soy phosphatidylcholine (HSPC) (in a molar ratio of 0.15:1:1.83) were dissolved in an organic solvent (methanol: chloroform solution in a ratio of 9:1 (v/v)). The mixed solution was evaporated in vacuo at 64°C to obtain the lipid film. Then add 2 mg/ml of DOX (dissolved in saline) into the resulting film, then ultrasonically vibrate for 20 min. Finally, put it into the probe-type ultrasonic for high-power ultrasonic for 20 minutes in ice bath, until a uniform supernatant is formed.

In vitro drug release: PDNPs were placed in a dialysis tube (MWCO, 3.5 kDa) and incubated in different pH buffers (pH 7.4, 6.5 or 5.0) at 37°C with a shaking speed of 110 rpm for 48 h. At presuppose time points, the sample solution was taken out and replaced by equal volume of the corresponding buffer solution. The loading rate and drug release content were detected through a microplate reader (Tecan SPARK-10M) (Ex: 505 nm, Em: 550 nm).

In vitro cytotoxicity: The cytotoxicity of the PDNPs against CT26 cells was assessed by MTT assay. Briefly, cells were initially seeded on a 96-well plate at a density of 1×10^4 cells/well and cultured overnight for attachment. Then, the cells were incubated with PDNPs and free DOX at series concentrations in different pH medium (pH 7.4 or pH 6.5) for 48 h. Afterwards, the culture medium was discarded and 100 μL of MTT stock solution (0.5 mg mL^{-1}) was added for another 4 h of incubation. The formed purple formazan crystals were dissolved in 100 μL of DMSO and the cell viability was determined by the UV absorbance at 480 nm detected by a microplate reader.

Cellular uptake: The cellular uptake assay was executed by flow cytometry (FCM) and confocal laser scanning microscope (CLSM, ZEISS LSM880). CT26 cells were seeded on a 24-well plate at a density of 4×10^4 cells/well and cultured overnight. The cells were treated with PDNPs (10 μg DOX mL^{-1}) in pH 7.4 or 6.5 medium for 0.5 h, 1 h, 2 h and 4 h, and the cells without treatment defined as control. Then, all cells were digested and resuspended in PBS for quantitative analysis using FCM. Moreover, the cellular uptake of PDNPs was observed using CLSM. After incubation with PDNPs for different time periods, the cells were stained with Hoechst 33342 for 5 min and visualized by CLSM.

Endosomal escape and disruption: CT26 cells were seeded on a 24-well plate at a density of 4×10^4 cells/well. After attachment, the cells were treated with PDNPs (10 μg DOX mL^{-1}) and incubated for 1 h, 4 h, 6 h and 8 h. Then, the cells were washed by PBS and stained with Lyso-Tracker Green DND26 for 30 min and Hoechst 33342 for 5 min, repectively. Afterwards, the samples were imaged by CLSM. The co-localization of lysosomes and

PDNPs were measured by Pearson correlation coefficient. The endosome disruption was evaluated using acridine orange (AO) staining. After treatment with PDNPs for 12 h, the CT26 cells was stained with AO stock for 30 min and then imaged by CLSM.

DNA damage: CT26 cells were seeded on a 24-well plate at a density of 4 × 10^4 cells/well. After attachment, the cells were incubated with free DOX and PDNPs for 24 h (10 μg DOX mL^{-1}). Then the cells were successively treated with 4% paraformaldehyde, 0.1% Triton X-100 and 5% bovine serum albumin. Afterwards, the cells were incubated with the anti-γ-H2AX and overnight at 4°C, then the cells were treated with Alexa Fluor 488-labeled Goat Anti-Rabbit IgG for 1 h in dark and DAPI for 5 min, respectively. All the samples were imaged by CLSM.

Intracellular trafficking: The intracellular pathway of PDNPs was tracked by TEM. Briefly, CT26 cells were incubated with 20 μg mL^{-1} of PDNPs for different time durations (4, 8 and 24 h) and were harvested by 0.25% trypsin. Then the cells were fixed by 2.5% glutaraldehyde solution and embedded in paraffin. Ultrathin slices (70 nm) were cut for TEM imaging.

ATP and LDH release: CT26 cells were seeded on 96-well plate at a density of 5 × 10^4 cells/well and cultured for attachment. Then the cells were exposed to Lipo-Dox, free DOX and PDNPs for 24 h. Then the cell supernatant was collected to determine the ATP or LDH release. The release amounts of ATP and LDH in the cell supernatant were measured by ATP Assay Kit and LDH Release Assay Kit, respectively.

In vitro HMGB1 release and CRT expression: CT26 cells seeded on 24-well plate at 4 × 10^4 cells/well were incubated with Lipo-Dox, free DOX or PDNPs (10 μg DOX mL^{-1}) for 24 h. The non-treated cells were served as control. After washing with PBS, the cells were fixed with 4% paraformaldehyde, permeabilized with 0.1% Triton X-100 and blocked with 5% BSA. Then the cells were treated with anti-HMGB1 or anti-CRT antibody and stored at 4°C overnight. Thereafter, the cells were incubated with Alexa Fluor 647-labeled and Alexa Fluor

488-labeled anti-rabbit secondary antibody respectively, followed by incubated with DAPI for nuclear staining. All the samples were observed by CLSM.

Western blot: The samples were lysed by RIRA reagent (Pierce, Rockford, IL, USA) and the supernatants were collected followed by centrifuging at 13,000 rpm for 10 min at 4°C to acquire the proteins. After protein quantification through the bicinchoninic acid method, 12% sodium dodecyl sulfate polyacrylamide gel electrophoresis (SDS-PAGE) was used for protein separation. Then the proteins were transferred onto polyvinylidene fluoride membranes (Millipore) and incubated with 5% skimmed milk for 1 h at room temperature. Afterwards, the proteins were incubated with primary antibodies against GSDME, Cleaved caspase-3 and β-actin at 4°C overnight and horseradish peroxidase (HRP) labeled secondary antibody at room temperature for 1 h. The protein blots were obtained using the ECL kit (Advansta) and the Odyssey system (LI-COR Biosciences) was used to visualize the protein band. The data were normalized to 1.

RNA-seq analysis: CT26 cells seeded on 12-well plate at 5×10^4 cells/well were treated with PDNPs (10 μg DOX mL^{-1}). After incubation for 24 h, cells were harvested for RNA extraction. The RNA-seq libraries were established and sequenced on Illumina NovaSeq 6000 platform.

Permeability in multicellular spheroids (MCSs): The CT26 MCSs tumour model were established as described in the previous report.[2] Upon the 3D MCSs model were set up, they were treated with PDNPs (10 μg DOX mL^{-1}) diluted with cultural medium of pH 7.4 or 6.5 and incubated for 6 h. Then the CT26 MCSs were shifted into a 12-well plate. After washing with PBS, the permeability of PDNPs in MCSs was observed by Z-stack images using CLSM.

In vivo biodistribution and intra-tumour penetration: BALB/c mice were administrated subcutaneously with 2.5×10^6 of CT26 cells at the right flank. After seven days, the mice were randomly divided into two groups and intravenously injected with DiR@PDNPs or free DiR (identical to 200μg DiR). The NIR images were captured at 6 h, 12 h, 24 h and 48 h

post-injection using a IVIS Lumina imaging system. The mice were sacrificed at 48 h, the tumours and major organs (heart, liver, spleen, lung and kidney) were extracted for *ex vivo* fluorescence distribution. The all images were quantified by the Living Image software. Then the tumours were subjected to frozen sectioned and observed the drug penetration by CLSM.

Pharmacokinetics: The S.D. rats were randomized into two groups and administrated with PDNPs and free DOX *via* tail vein injection. At presuppose time points, the blood samples were obtained from eye socket. Then, the plasma was collected *via* centrifuging the blood samples at 4°C. Afterwards, the obtained plasma was co-incubated with methanol, and shaking for 1 h at speed of 110 rpm to precipitate protein. The drug concentration in plasma was then measured by microplate reader (Ex: 505 nm, Em: 550 nm).

In vivo anti-tumour immunity: The CT26 or 4T1 tumour-bearing BALB/c mice models were established by the aforementioned method. The mice were randomly divided into four groups when the tumour volume reached ~100 mm^3, and then treated with saline, Lipo-Dox, free DOX or PDNPs (dose of 5 mg kg^{-1}) through tail vein injection every three days for three times. The tumour volume and body weight were monitored every day after injection. The tumour volume was determined as [(length × $width^2$) / 2].

On the second day after the last treatment, the spleens of CT26 tumour-bearing mice were extracted and isolated to single-cell suspension. The single-cell suspension of spleens was obtained through the density gradient centrifugation method. Then the single-cell suspension samples were stained with antibodies, including anti-CD45-PC5.5, anti-CD11c-FITC, anti-MHC II-PC7, anti-CD80-PE, anti-CD86-APC, anti-CD3-FITC, anti-CD4-ef450, anti-CD8-PC5.5, anti-CD44-PE, anti-CD62L-APC, anti-CD11b-FITC, anti-Ly6C-APC, anti-Ly6G-PE. The flow cytometry resulting was acquired using CytExpert software (V2.0) and the data were analyzed using Flow Jo software (Tree Star, V10.6.2). The gating strategy is presented in Figure S38-40.

The residual tumours were subjected to frozen sectioned for immunofluorescence and immunohistochemical staining. The immunostained method was as described in the previous report.[3] After the treatments, anti-CD86 mixed with anti-CD11c, anti-CD8α mAb mixed with anti-PD-1 mAb, anti-CD103 mixed with anti-CD11c, anti-CD103 mixed with anti-CD8 were detected with immunofluorescence staining. The CD86, CD8, CD103, CD11c, Arginase-1, Granzyme B and Cleaved caspase-3 were conducted by immunohistochemistry staining scanned by 3DHISTECH (panoramic MIDI).

The combination therapy of PDNPs with anti-PD-1 antibody: CT26 and 4T1 tumour-bearing BALB/c mice were randomly grouped (n = 5) and administrated with saline, anti-PD-1, PDNPs or anti-PD-1 + PDNPs, repectively. The therapeutic schedules were the same as the above mentioned, and anti-PD-1 (200 μg per mouse) was treated by intraperitoneal administration one day after the injection of PDNPs. The tumour volume and body weight were recorded every day during the treatment process, and the tumour growth was monitored through bright filed and bioluminescence imaging at 1 day, 7 days, 25 days, 35 days and 45 days. The mice were considered as euthanized when the tumour diameter exceeded 2 cm at any direction.

Biosafety Evaluation: 6-week-old female BABL/c mice were divided into four groups and then administrated with saline, Lipo-Dox, free DOX, and PDNPs (dose of 5 mg kg^{-1}). On days 1, 7 and 14, the blood samples were obtained from eye socket and routine blood tests, including white blood cell (WBC), lymphocytes ratio (Lymph), monocyte (Mon), granulocyte (Gran), red blood cell count (RBC), hemoglobin (HGB), blood-cell-specific volume (HCT), mean corpuscular volume (MCV), mean corpuscular hemoglobin content (MCH), mean corpuscular-hemoglobin concentration (MCHC), red cell volume distribution width (RDW), blood platelet counts (PTL), mean platelet volume (MPV), platelet distribution width (PDW) and procalcitonin (PCT) were conducted on a hematology analyzer (Mindray BC-2600Vet).

Statistical Analysis: All values were expressed as mean ± standard deviation (SD). For normally distributed data sets with equal variances, Student's t-test was used to analyze the significance difference between two groups, one-way ANOVA testing was used to analyze the significance difference among several groups. Upon Brown-Forsythe test, the *P*-value was more than 0.05 for each figure. Then Tukey's multiple comparisons test for post hoc test was followed to compare the mean of each column with the mean of every other column. In all cases, statistical significance was defined as $^{*}P < 0.05$, $^{**}P < 0.01$ and $^{***}P < 0.001$. All the graphing and statistical analysis were carried out using Prism (V8; Graphpad).

References

[1] X. Guan, Z. Guo, L. Lin, J. Chen, H. Tian, X. Chen, *Nano Letters* **2016**, 16, 6823.
[2] T. Zhang, H. Xiong, X. Ma, Y. Gao, P. Xue, Y. Kang, Z.-J. Sun, Z. Xu, *Small Methods* **2021**, 5, 2100115.
[3] X. Ma, S. Yang, T. Zhang, S. Wang, Q. Yang, Y. Xiao, X. Shi, P. Xue, Y. Kang, G. Liu, Z.-J. Sun, Z. Xu, *Acta Pharmaceutica Sinica B* **2022**, 12, 451.

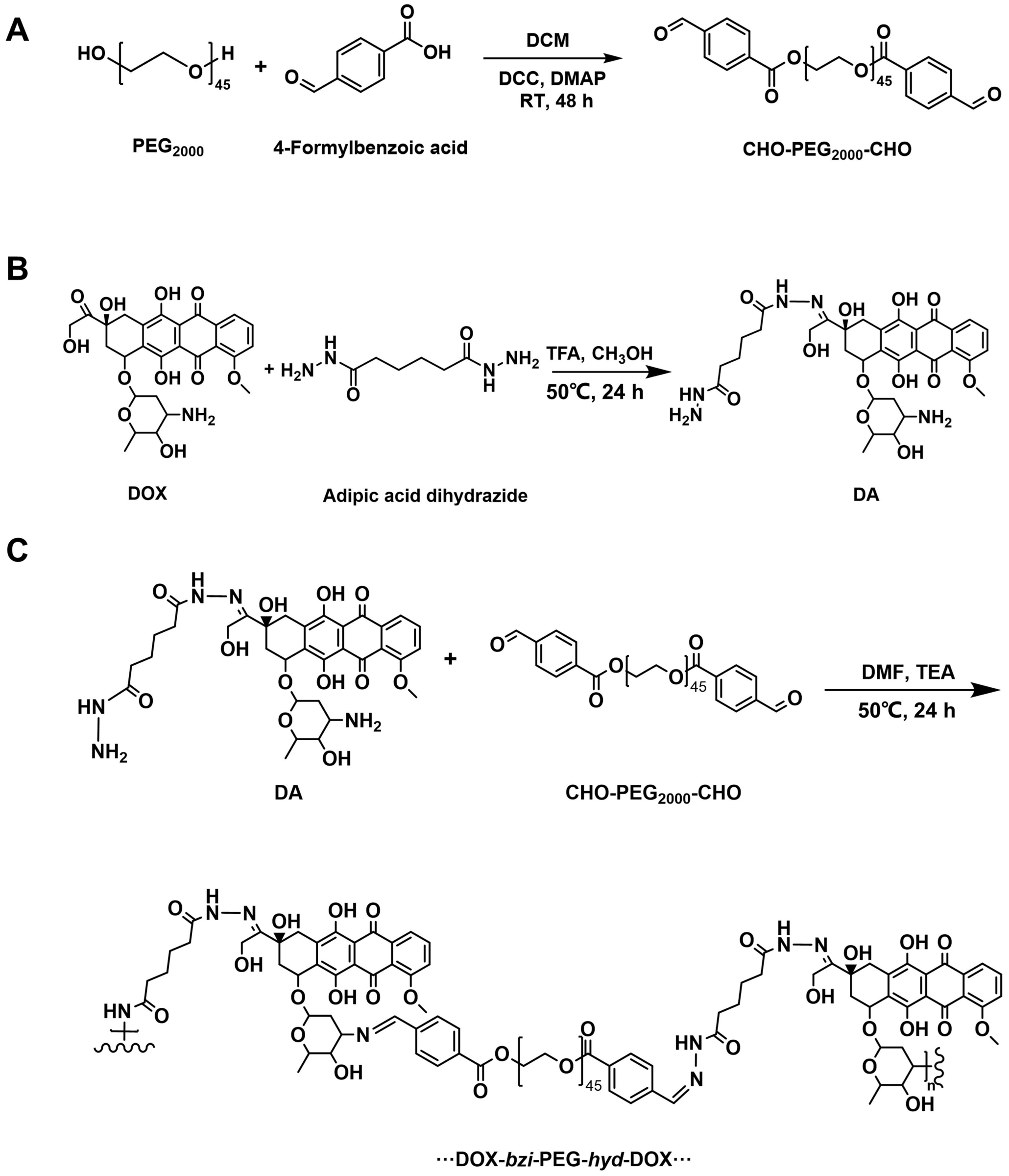

Figure S1. Synthetic routes of (A) CHO-PEG-CHO, (B) DA, and (C) PDNPs.

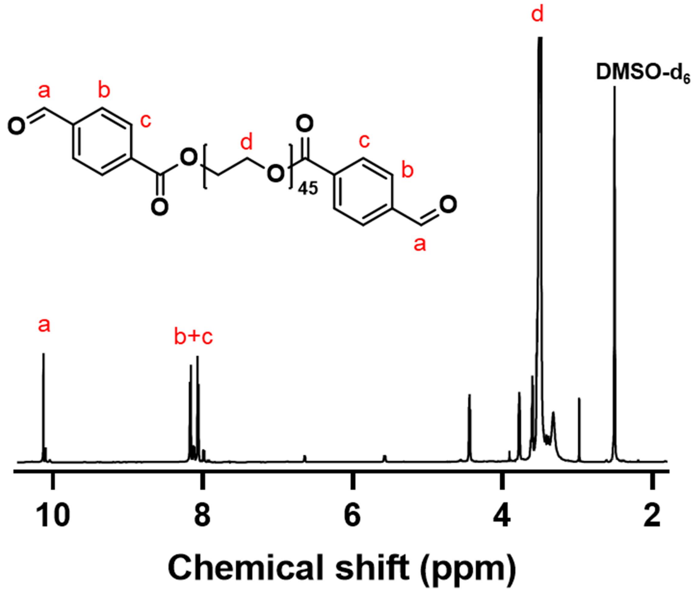

Figure S2. ^{1}HNMR spectroscopy of CHO-PEG-CHO in DMSO-*d₆*.

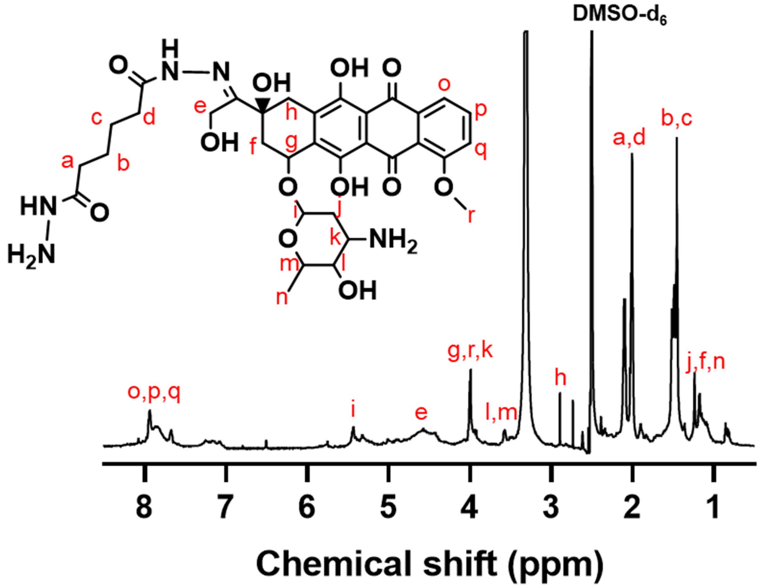

Figure S3. ^{1}HNMR spectroscopy of DA in DMSO-d_6.

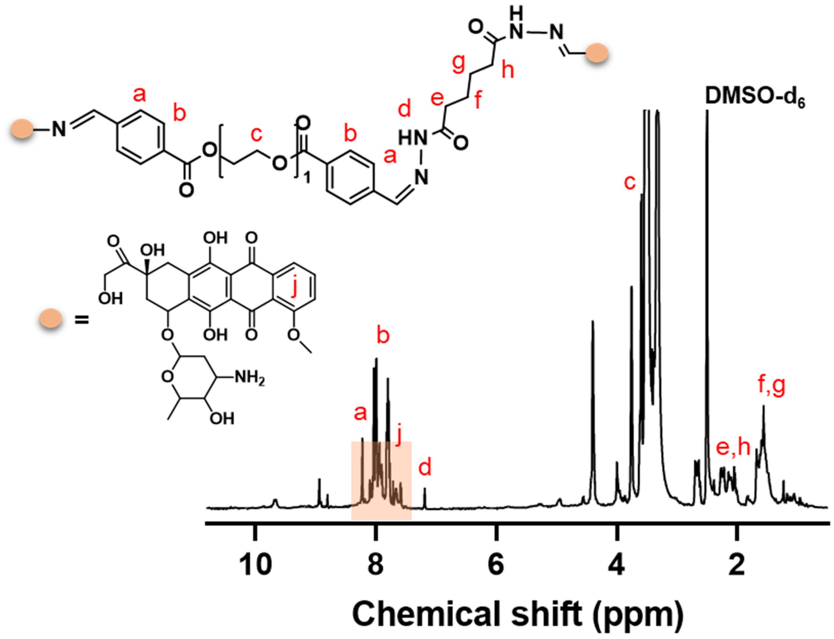

Figure S4. ^{1}HNMR spectroscopy of PDNPs in DMSO-d_6.

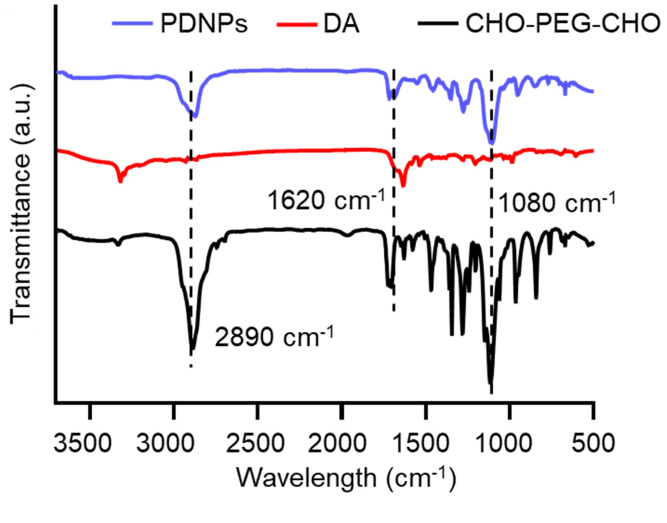

Figure S5. FTIR spectra of CHO-PEG-CHO, DA and PDNPs.

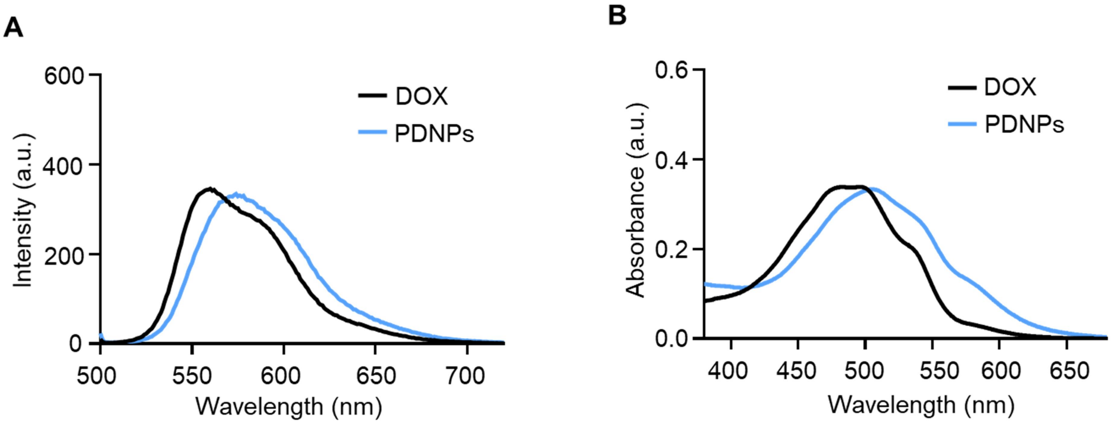

Figure S6. (A) Fluorescence spectra and (B) UV-vis absorption spectra of DOX and PDNPs.

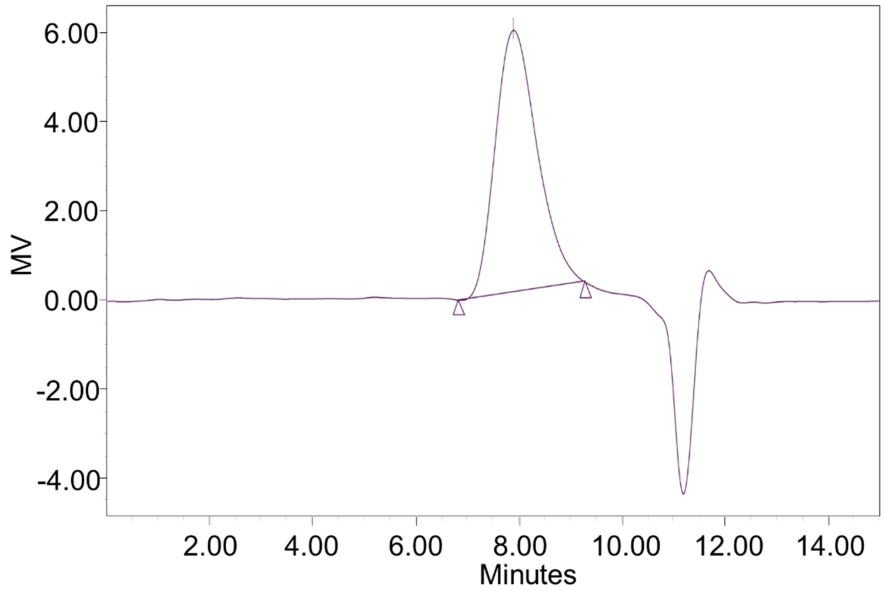

Polymer	Mn (Daltons)	Mw (Daltons)	Mz (Daltons)	Polydispersity	Mz/Mw
PDNPs	1.2×10^4	2.6×10^4	4.4×10^4	2.1	1.7

Figure S7. Gel Permeation Chromatograph (GPC) data of PDNPs in DMF.

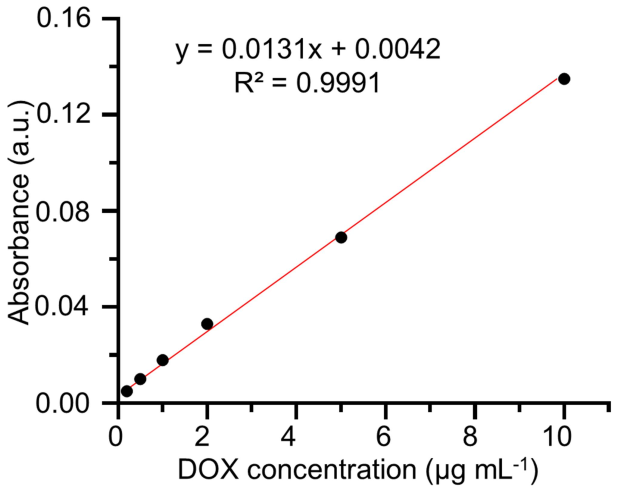

Figure S8. The calibration curves of DOX determined by UV-vis spectrometer.

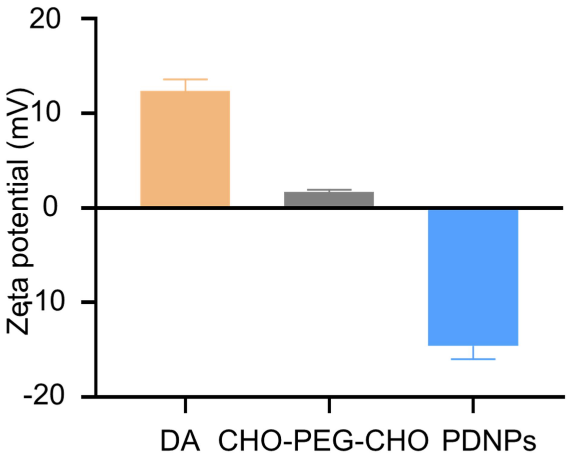

Figure S9. Zeta potentials of DA, CHO-PEG-CHO and PDNPs (Data presented as mean ± SD, n = 3).

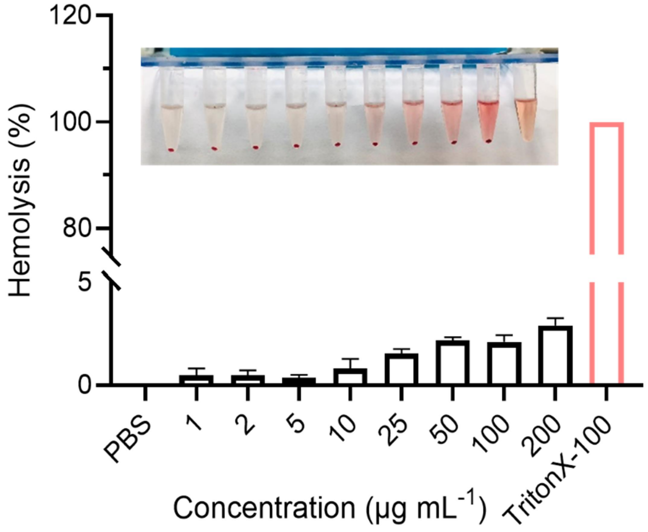

Figure S10. Haemolysis rates and photographs of PDNPs at different DOX concentrations (Data presented as mean ± SD, n = 3).

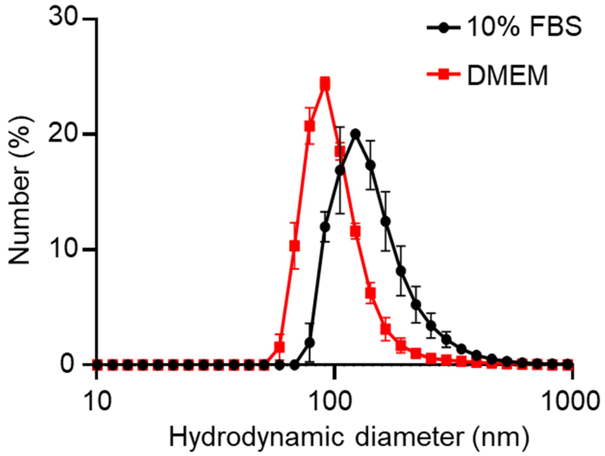

Figure S11. Hydrodynamic distribution of PDNPs incubated with PBS containing 10% FBS and DMEM at 48 h (Data presented as mean ± SD, n = 3).

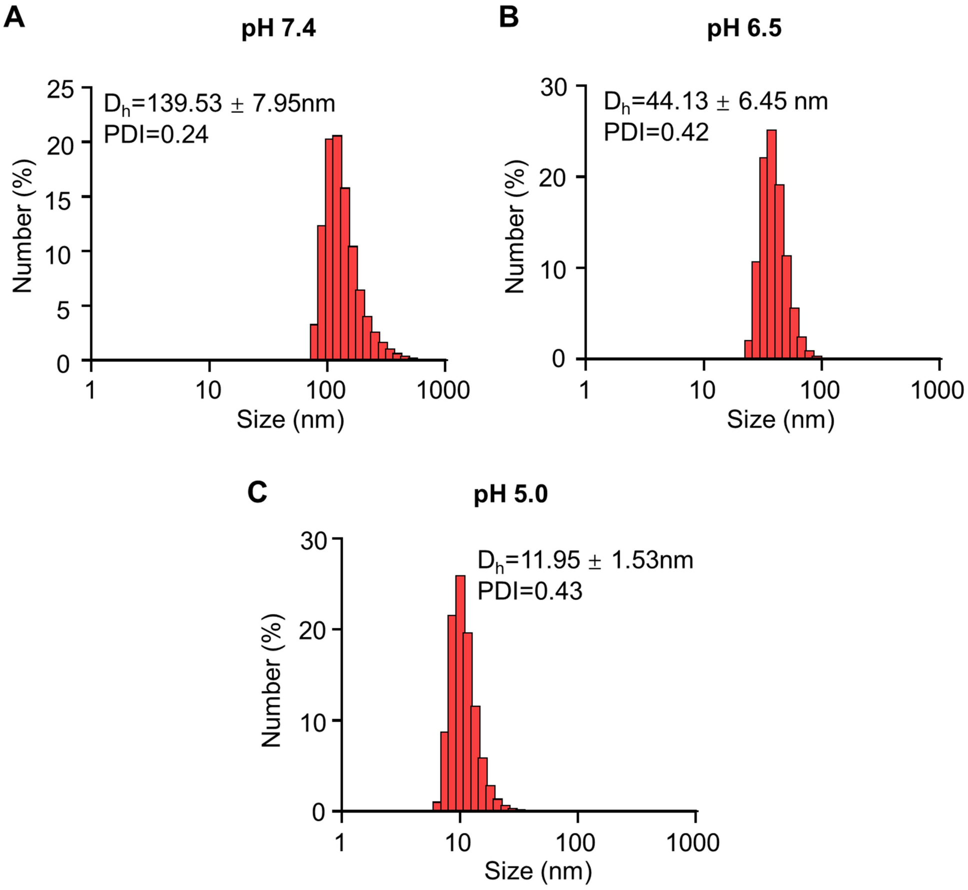

Figure S12. Hydrodynamic distribution of PDNPs at (A) pH 7.4, (B) pH 6.5 or (C) pH 5.0.

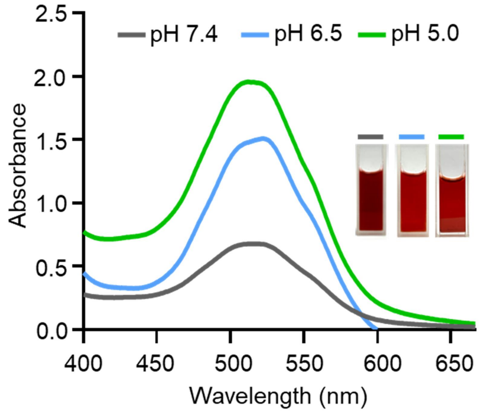

Figure S13. UV-vis absorption spectrum of PDNPs incubated in medium at pH 7.4, 6.5 or 5.0.

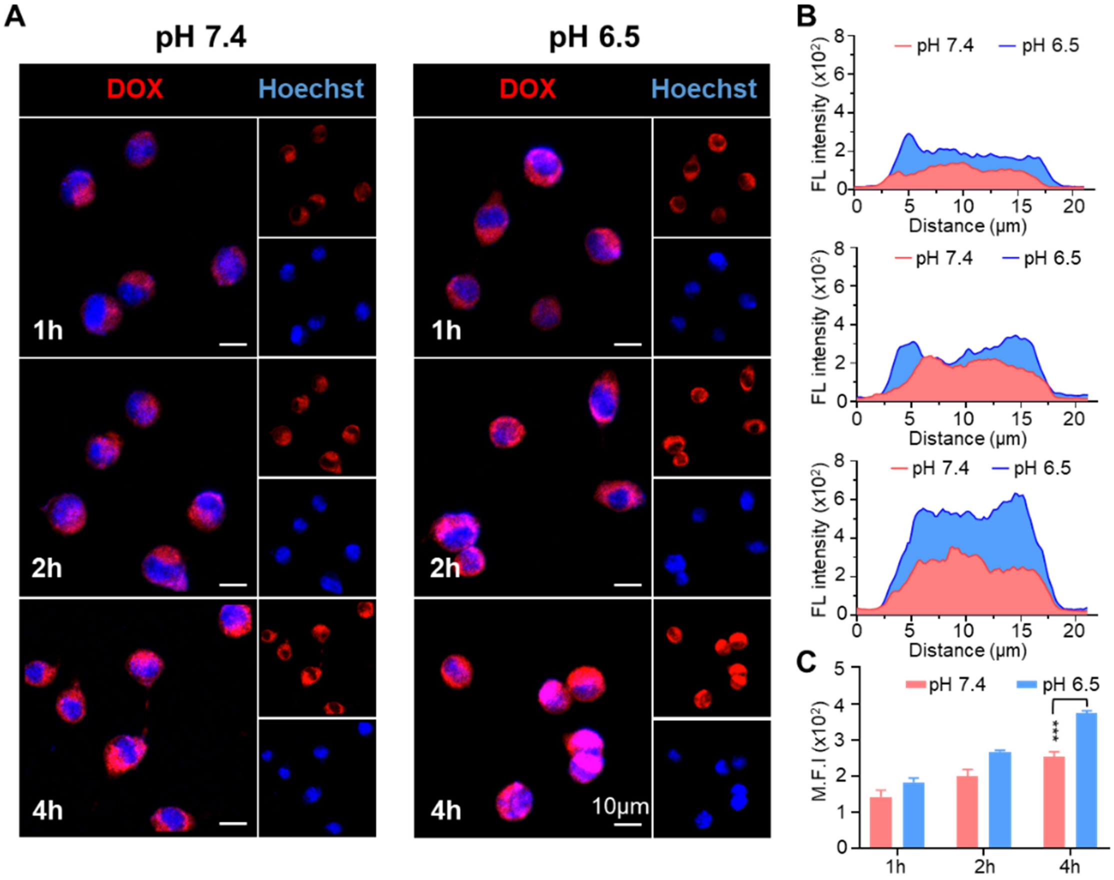

Figure S14. (A) CLSM images, (B) fluorescence (FL) intensity profiles, and (C) mean fluorescence intensity of CT26 cells incubated with PDNPs for 1 h, 2 h and 4 h at pH 7.4 or 6.5 (Data presented as mean ± SD, n = 3, *P*-values are calculated using Student's t-test, $^{***}P < 0.001$).

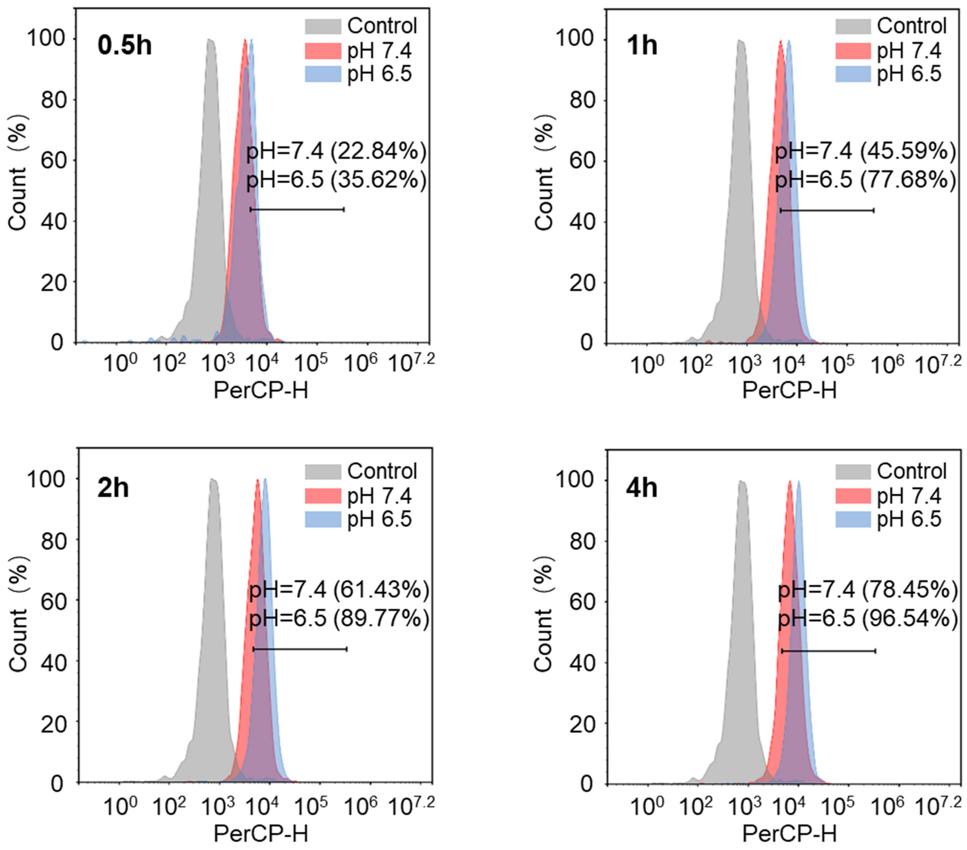

Figure S15. Flow cytometric analysis of intracellular uptake in CT26 cells incubated with PDNPs for 0.5 h, 1 h, 2 h and 4 h at pH 7.4 or 6.5.

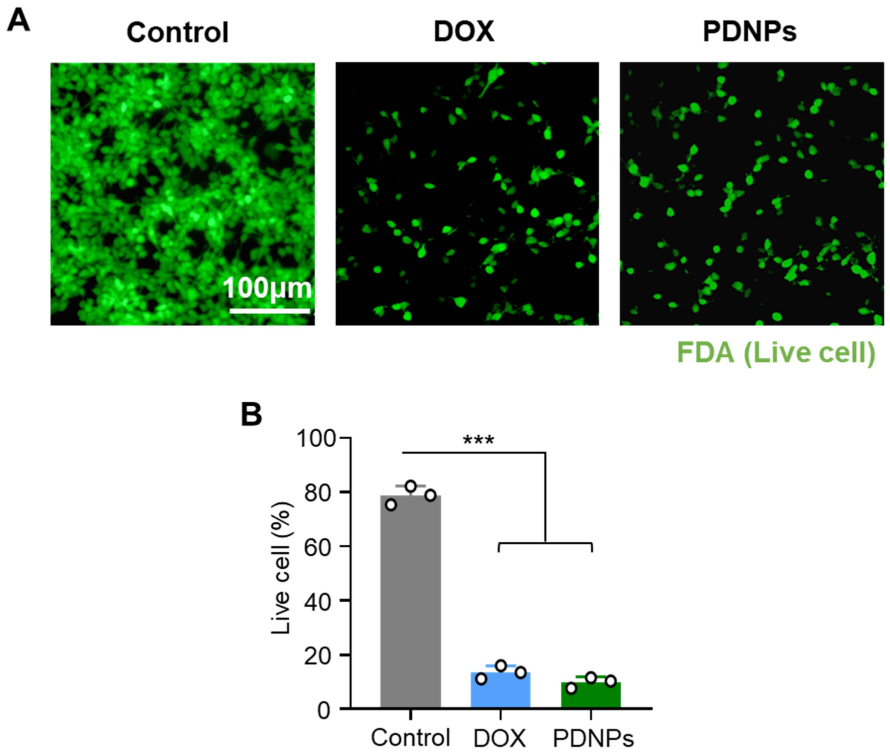

Figure S16. Fluorescence images and quantification of CT26 cells treated with PBS, DOX, PDNPs for 24 h (Data presented as mean ± SD, n = 3, P-values are calculated using one-way ANOVA, ***$P < 0.001$).

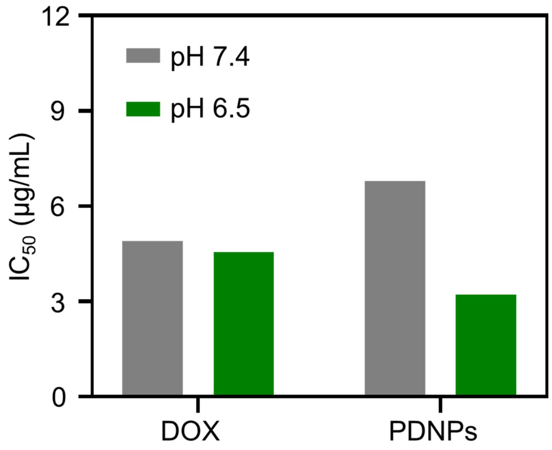

Figure S17. The IC_{50} values of DOX and PDNPs in CT26 cells at pH 7.4 or 6.5.

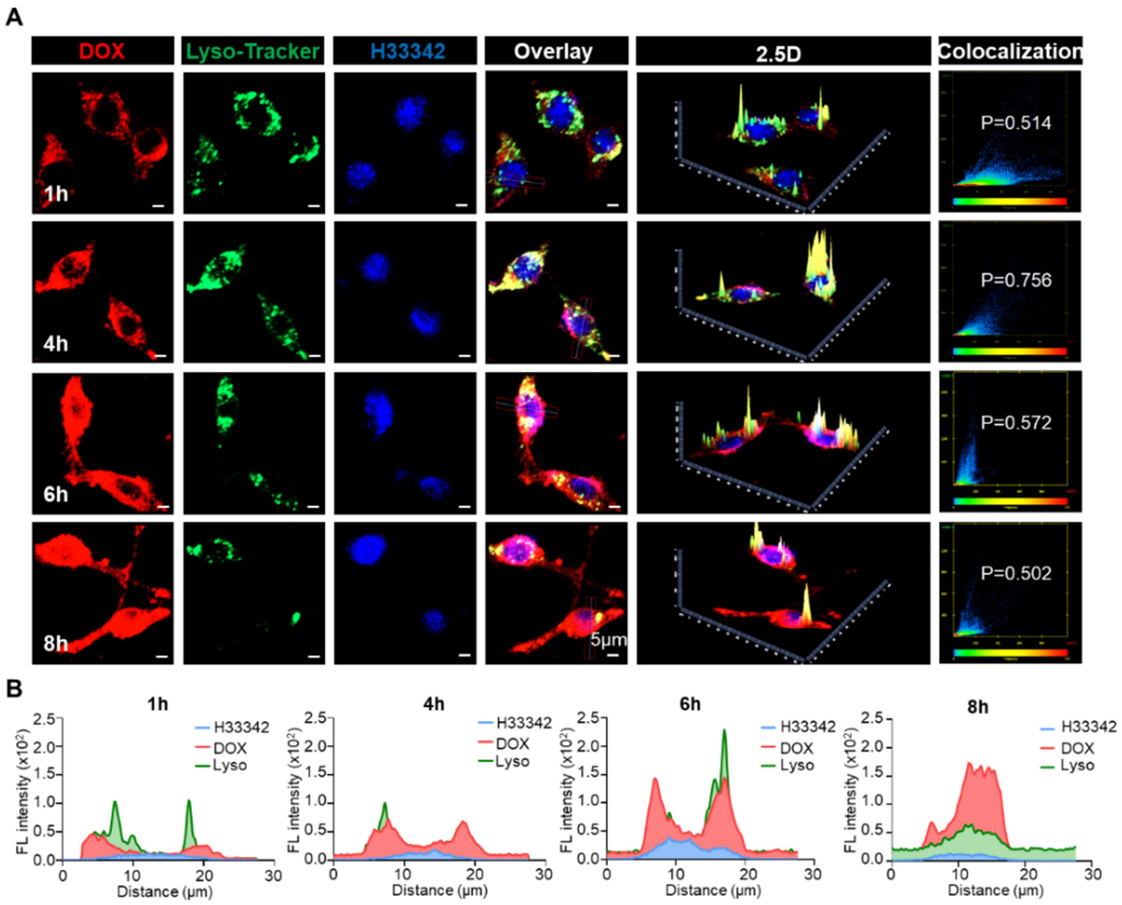

Figure S18. (A) Co-localization images and (B) FL intensity profiles of CT26 cells stained with Lyso-Tracker green upon incubation with PDNPs for 1 h, 4 h, 6 h and 8 h.

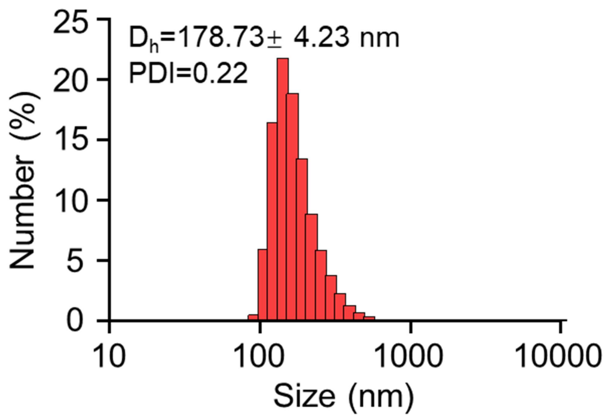

Figure S19. Hydrodynamic distribution of Lipo-Dox.

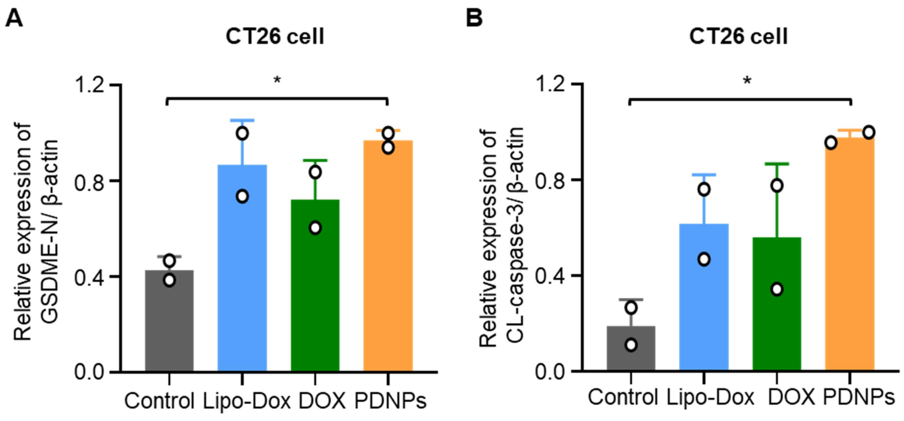

Figure S20. (A) The relative GSDME-N expression levels and (B) Cleaved caspase-3 (CL-caspase-3) expression levels in CT26 cells (Data presented as mean ± SD, n = 2, *P*-values are calculated using one-way ANOVA, *$P < 0.05$, **$P < 0.01$).

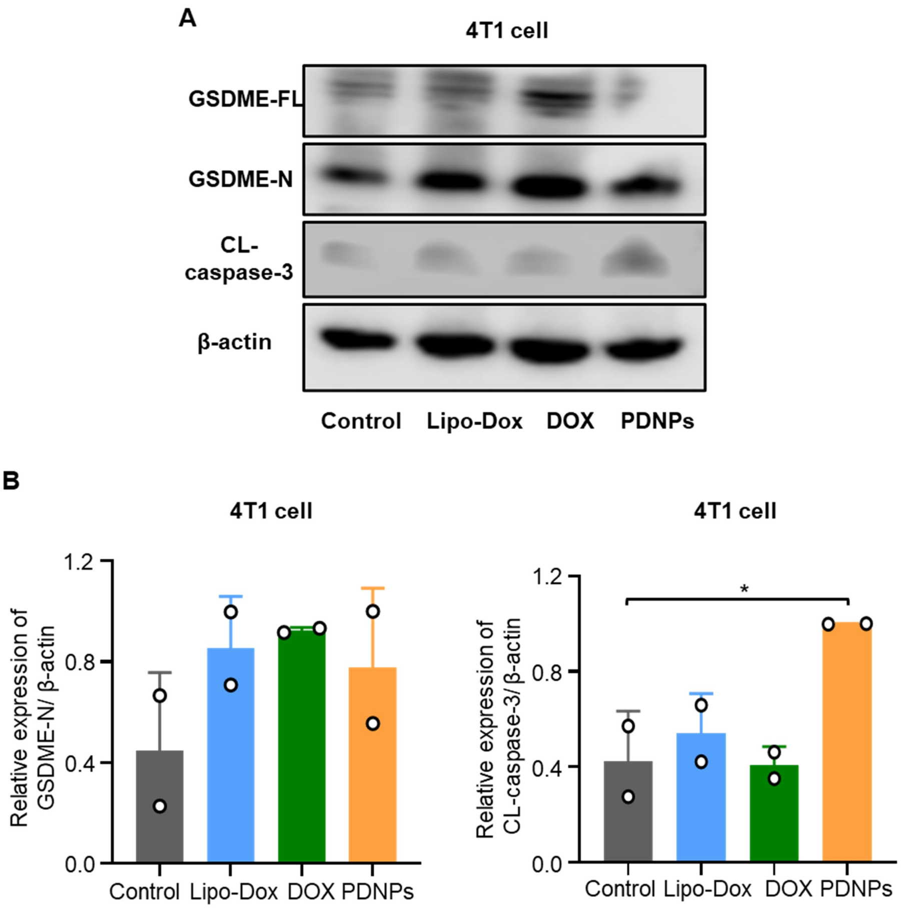

Figure S21. (A) Western blotting detection of GSDME-FL, GSDME-N terminal, CL-caspase-3 in 4T1 cells upon PDNPs treatment. (B) The relative expression levels of GSDME-N and CL-caspase-3 in 4T1 cells (Data presented as mean ± SD, n = 2, *P*-values are calculated using one-way ANOVA, $^{*}P < 0.05$, $^{**}P < 0.01$).

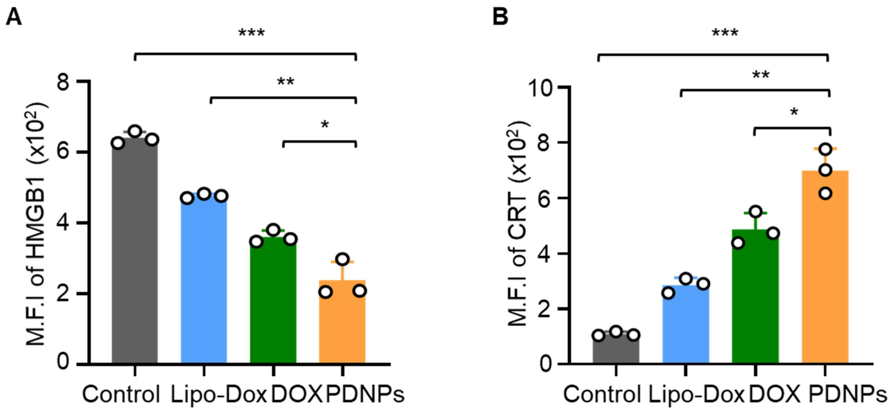

Figure S22. The quantitative analysis of (A) HMGB1 secretion and (B) CRT expression in CT26 cells (Data presented as mean ± SD, n = 3, *P*-values are calculated using one-way ANOVA, *$P < 0.05$, **$P < 0.01$, ***$P < 0.001$).

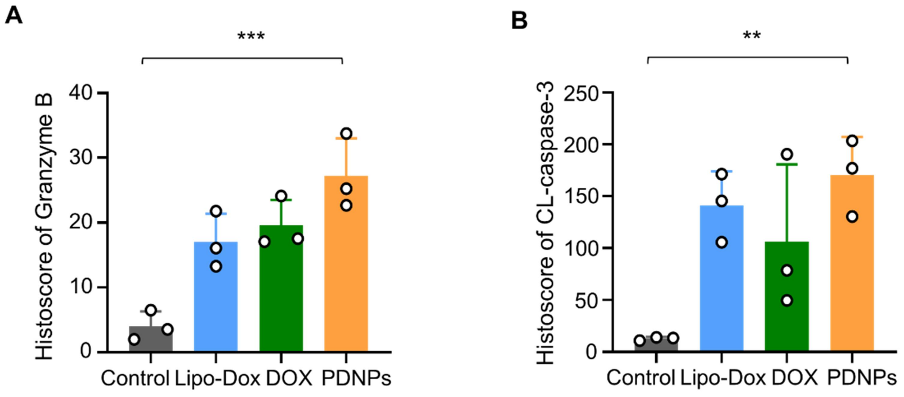

Figure S23. The quantitative analysis of immunohistochemical of (A) Granzyme B and (B) CL-caspase-3 in CT26 tumour tissues (Data presented as mean ± SD, n = 3, *P*-values are calculated using one-way ANOVA, **$P < 0.01$, ***$P < 0.001$).

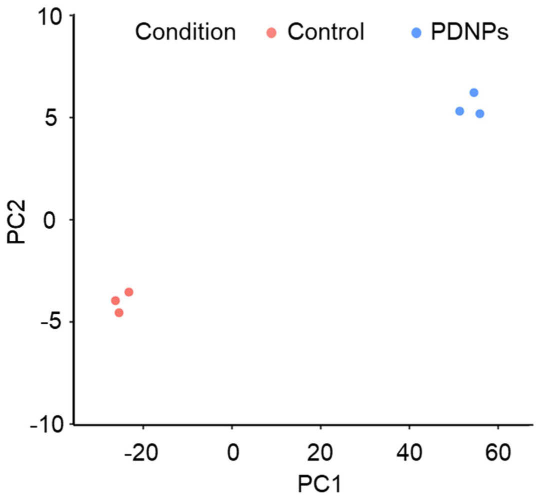

Figure S24. Principal component analysis (PCA) for the gene expression profile correlations between different groups (Data presented as mean ± SD, n = 3).

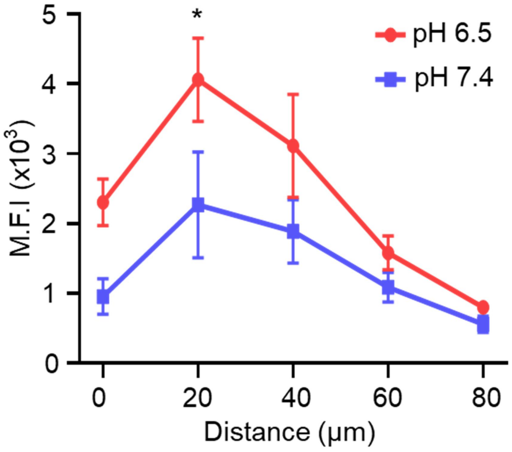

Figure S25. Quantitation of CT26 MCSs at varied depths upon incubation with PDNPs for 6 h at pH 7.4 or 6. (Data presented as mean ± SD, n = 3, *P*-values are calculated using Student's t-test, $^*P < 0.05$).

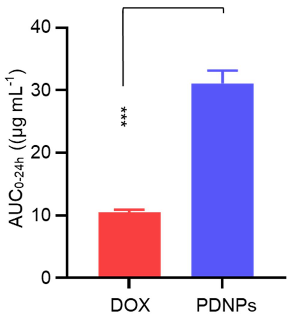

Figure S26. The area under the concentration-time curve (AUC_{0-24}) of PDNPs and free DOX (Data presented as mean ± SD, n = 3, *P*-values are calculated using Student's t-test, ***$P <$ 0.001).

Parameters	DOX	PDNPs
$t_{1/2}$ (h)	1.33±0.04	4.98±0.95
AUC_{0-t} (μg mL^{-1} * h)	10.52±0.39	31.17±2.142

Table S1. Pharmacokinetics parameters of DOX and PDNPs in S.D. rats (Data presented as mean ± SD, n = 3).

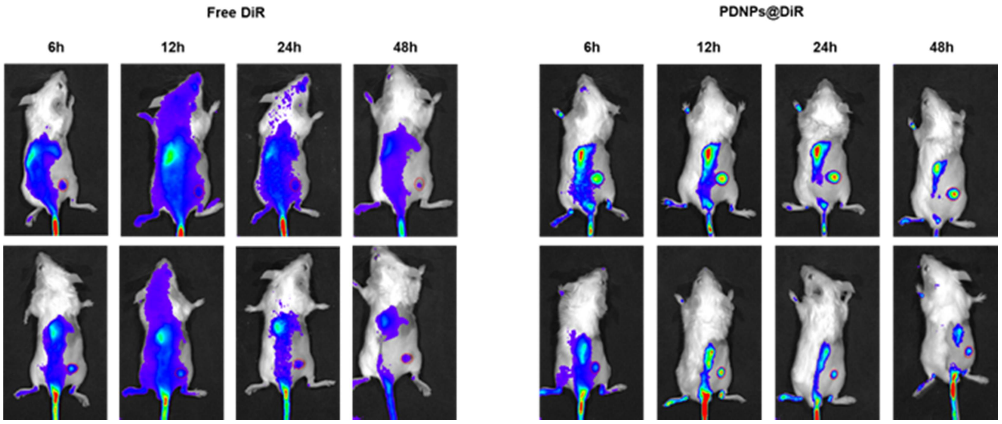

Figure S27. *In vivo* NIR imaging of free DiR and PDNPs@DiR in CT26-bearing mice. Red circles denote the tumour region (Data presented as mean ± SD, n = 3).

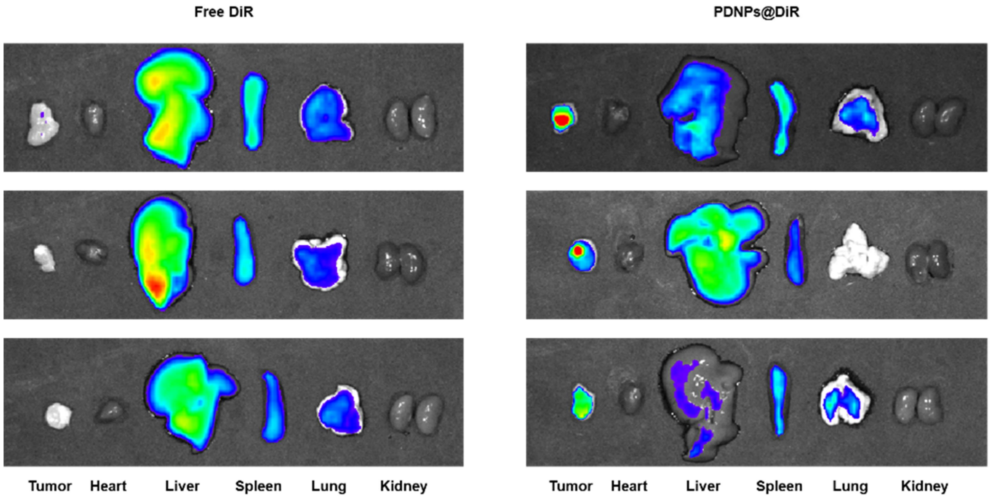

Figure S28. The fluorescence images of the excised tumours and organs at 48 h postinjection (Data presented as mean ± SD, n = 3).

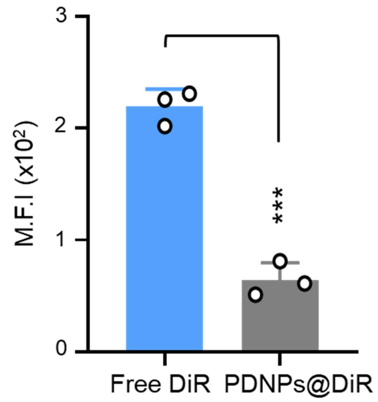

Figure S29. Quantitative analysis of PDNPs in the slices of CT26 tumour tissue at 48 h postinjection (Data presented as mean ± SD, n = 3, *P*-values are calculated using Student's t-test, ****P* < 0.001)..

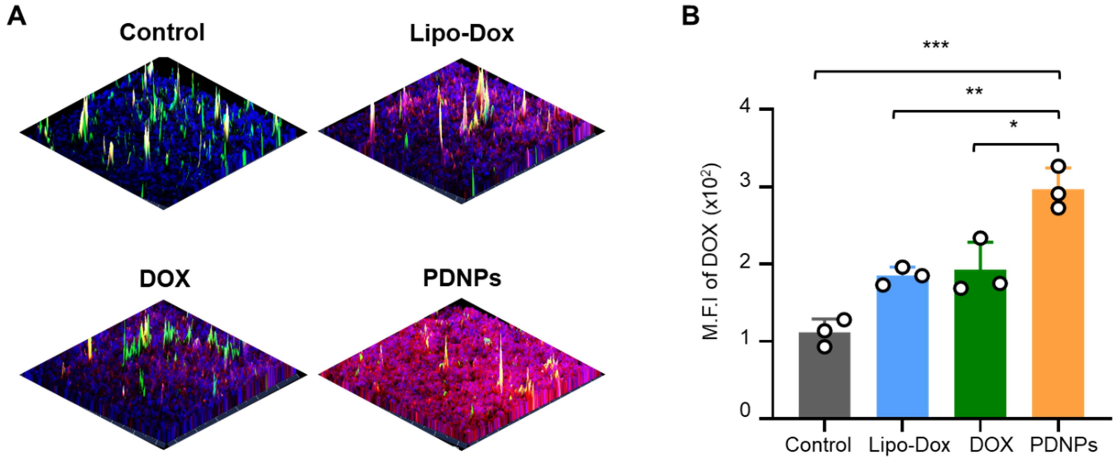

Figure S30. (A) The 2.5D images and (B) quantitative analysis of drug penetration in CD31 immunofluorescence staining assay (Data presented as mean ± SD, n = 3, *P*-values are calculated using one-way ANOVA, **P* < 0.05, ***P* < 0.01, ****P* < 0.01).

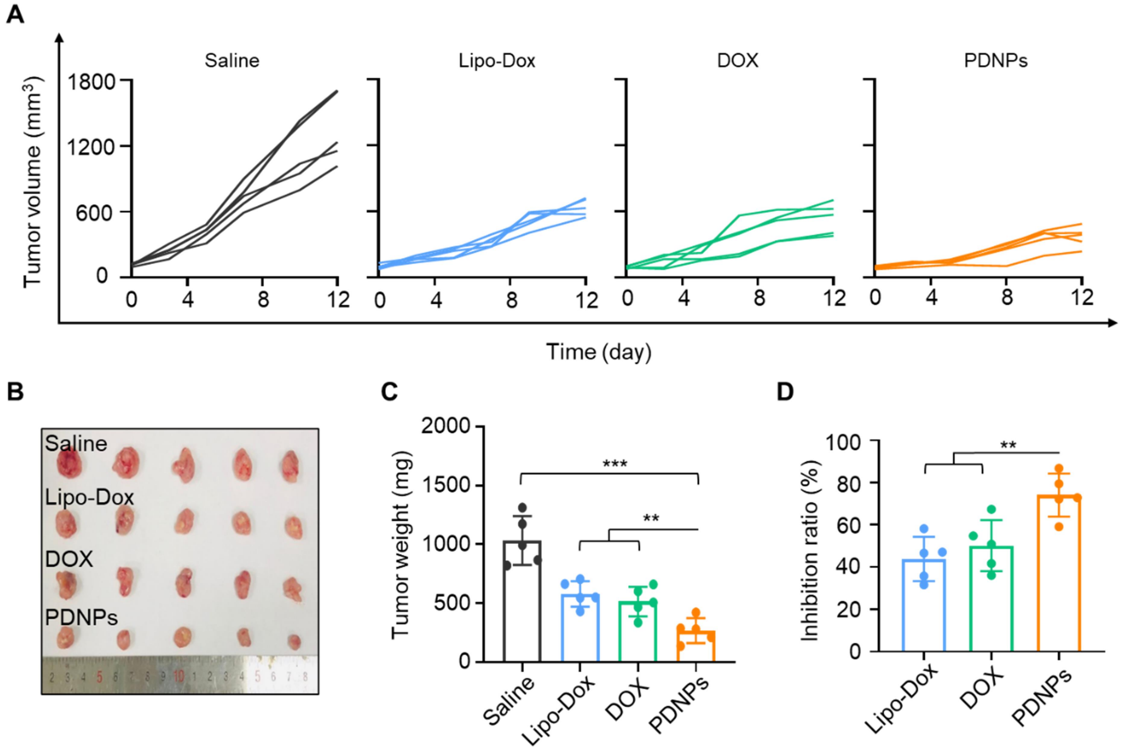

Figure S31. (A) Individual tumour growth curves of CT26 tumours in different treatment groups. (B) Tumour images and (C) the corresponding weight. (D) The tumour inhibition ratio of different treatment groups versus control (Data presented as mean ± SD, n = 5, *P*-values are calculated using one-way ANOVA, $^{**}P < 0.01$, $^{***}P < 0.01$).

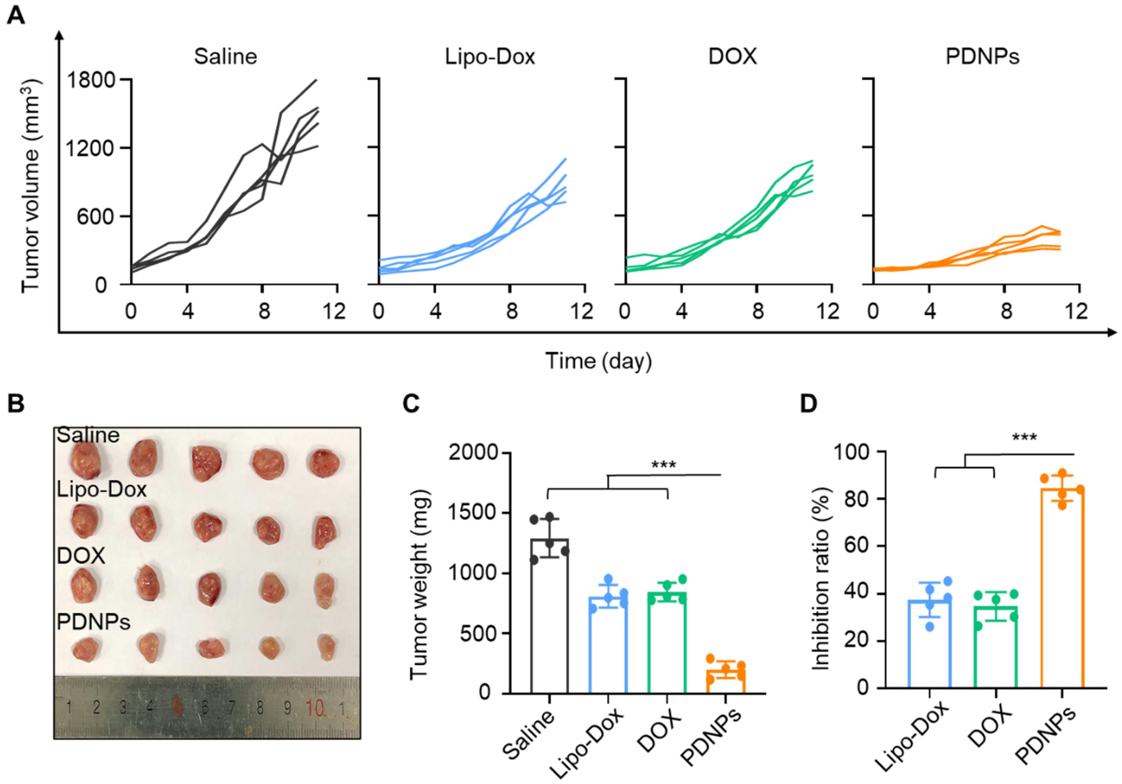

Figure S32. (A) Individual tumour growth curves of 4T1 tumours in different treatment groups. (B) Tumour images and (C) the corresponding weight. (D) The tumour inhibition ratio of different treatment groups versus control (Data presented as mean ± SD, n = 5, *P*-values are calculated using one-way ANOVA, ***$P < 0.01$)..

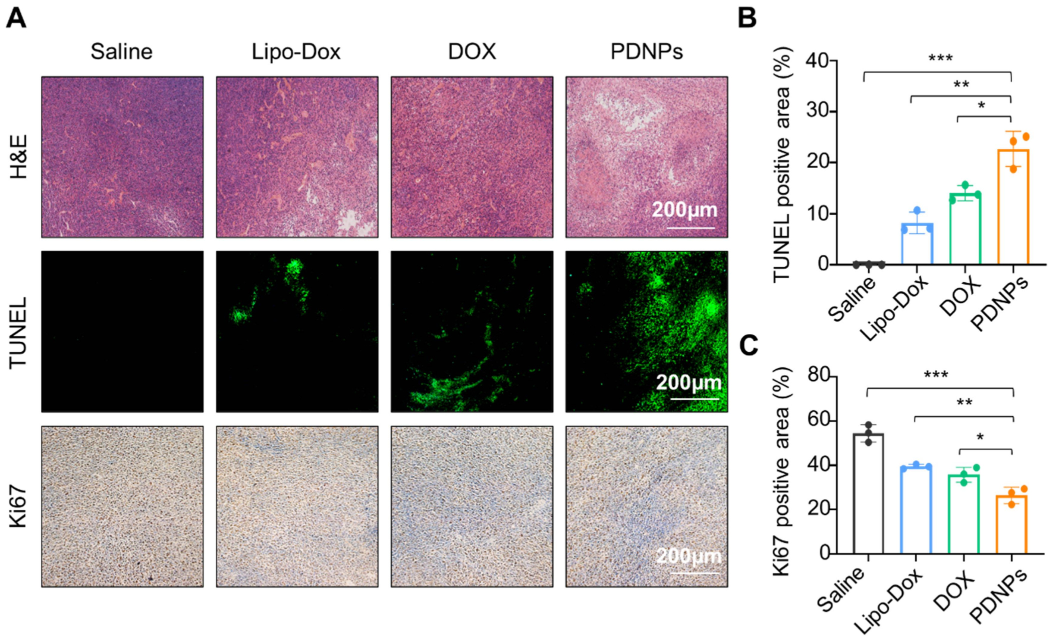

\

Figure S33. (A) H&E, TUNEL and Ki67 staining of CT26 tumour slices collected from the mice treated with different groups. (B) Relative apoptotic tumour cells in different groups. (C) Relative proliferative tumour cells in different groups (Data presented as mean ± SD, n = 3, *P*-values are calculated using one-way ANOVA, $*P < 0.05$, $**P < 0.01$, $***P < 0.01$).

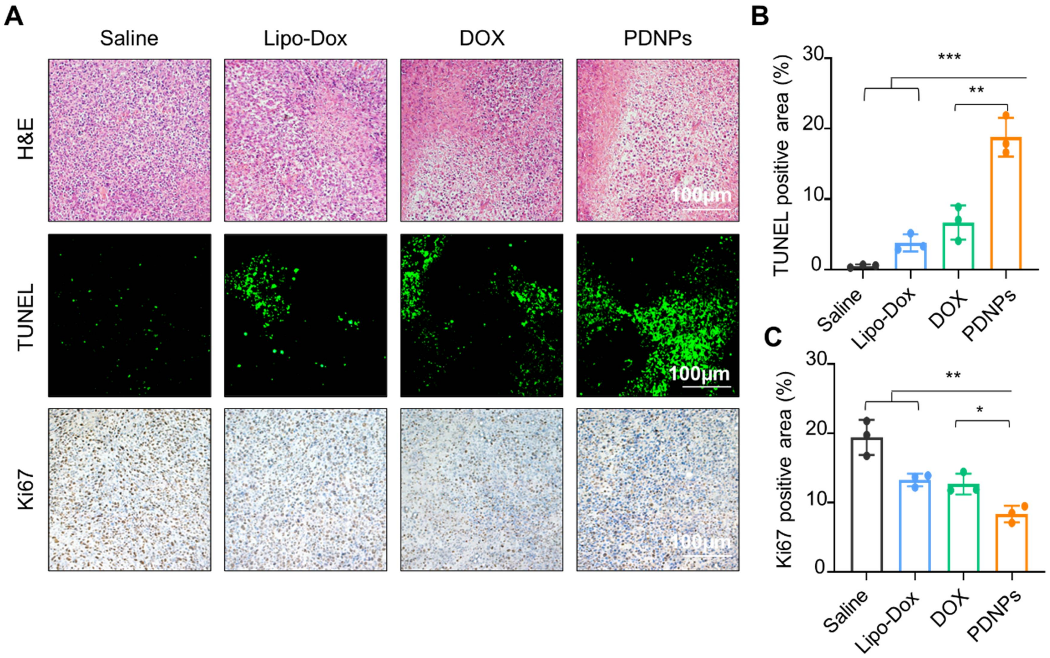

Figure S34. (A) H&E, TUNEL and Ki67 staining of 4T1 tumour slices collected from the mice treated with different groups. (B) Relative apoptotic tumour cells in different groups. (C) Relative proliferative tumour cells in different groups (Data presented as mean ± SD, n = 3, *P*-values are calculated using one-way ANOVA, *$P < 0.05$, **$P < 0.01$, ***$P < 0.01$).

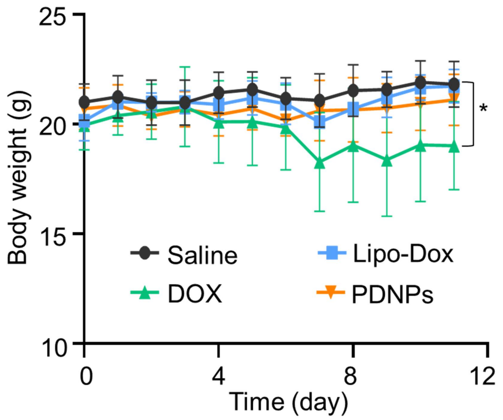

Figure S35. Body weight change of the CT26 tumour-bearing mice measured during the treatment period (Data presented as mean ± SD, n = 5, P-values are calculated using one-way ANOVA, *$P < 0.05$).

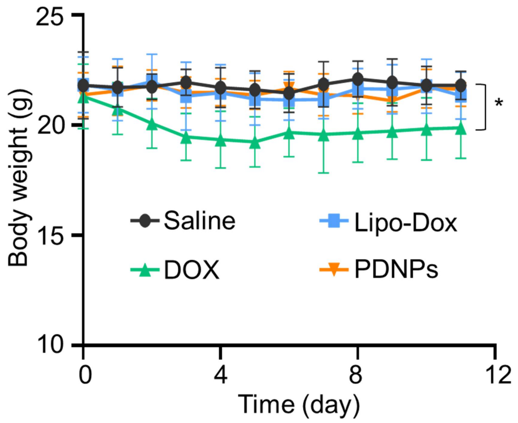

Figure S36. Body weight change of the 4T1 tumour-bearing mice measured during the treatment period (Data presented as mean ± SD, n = 5, P-values are calculated using one-way ANOVA, *$P < 0.05$).

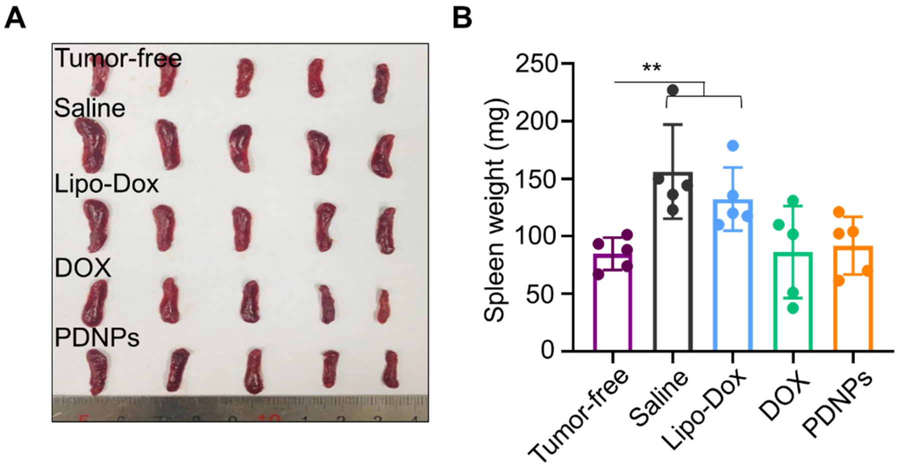

Figure S37. (A, B) The spleen images of tumour-free and CT26 tumour-bearing mice and the corresponding weight (Data presented as mean ± SD, n = 5, *P*-values are calculated using one-way ANOVA, ***P* < 0.01).

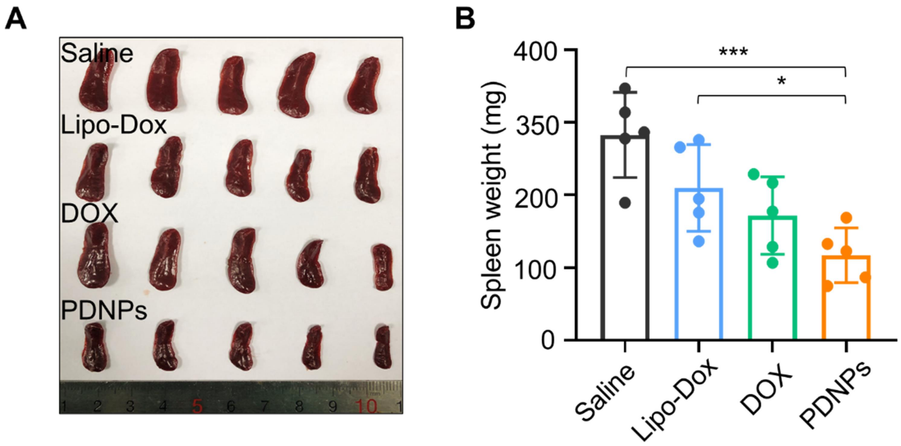

Figure S38. (A, B) The spleen images of 4T1 tumour-bearing mice and the corresponding weight (Data presented as mean ± SD, n = 5, *P*-values are calculated using one-way ANOVA, **P* < 0.05, ****P* < 0.001).

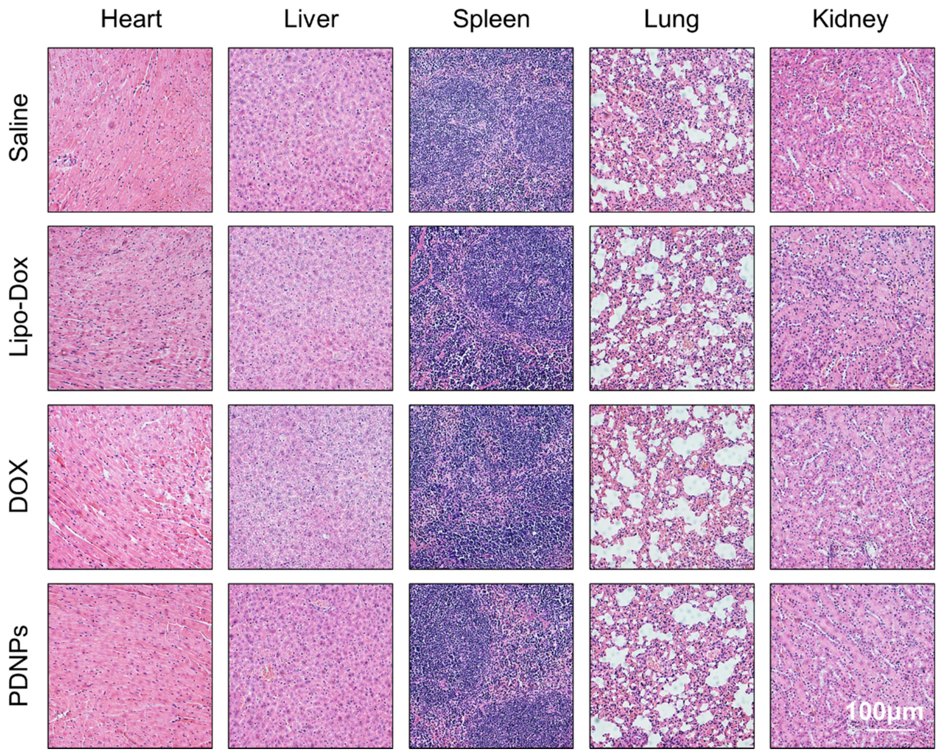

Figure S39. The representative H&E staining slices of major organs collected from the CT26 tumour-bearing mice after different treatments.

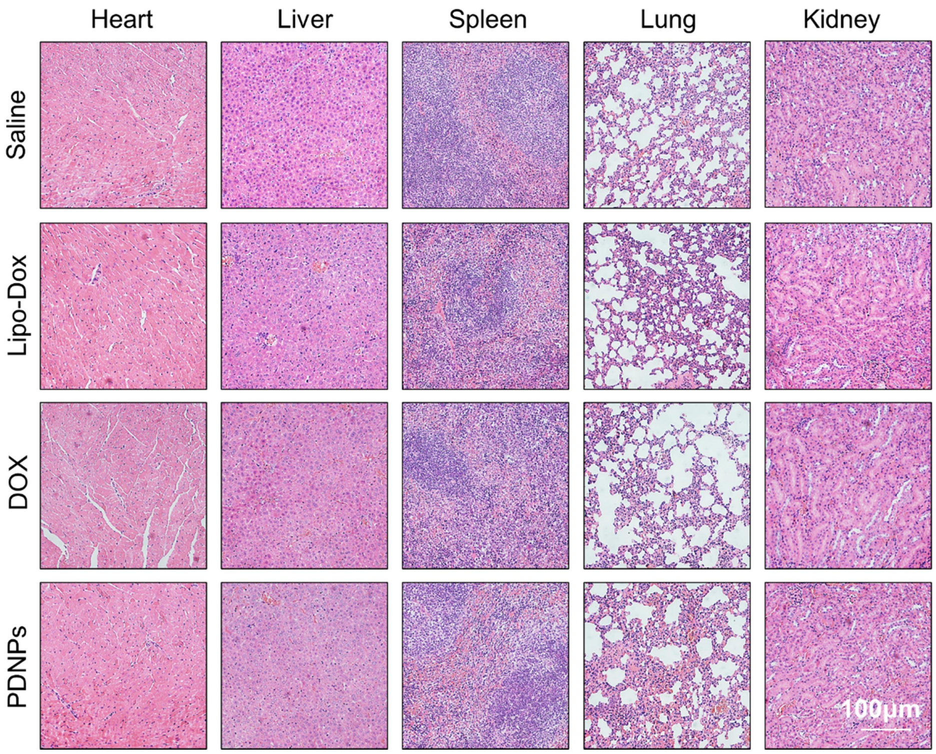

Figure S40. The representative H&E staining slices of major organs collected from the 4T1 tumour-bearing mice after different treatments.

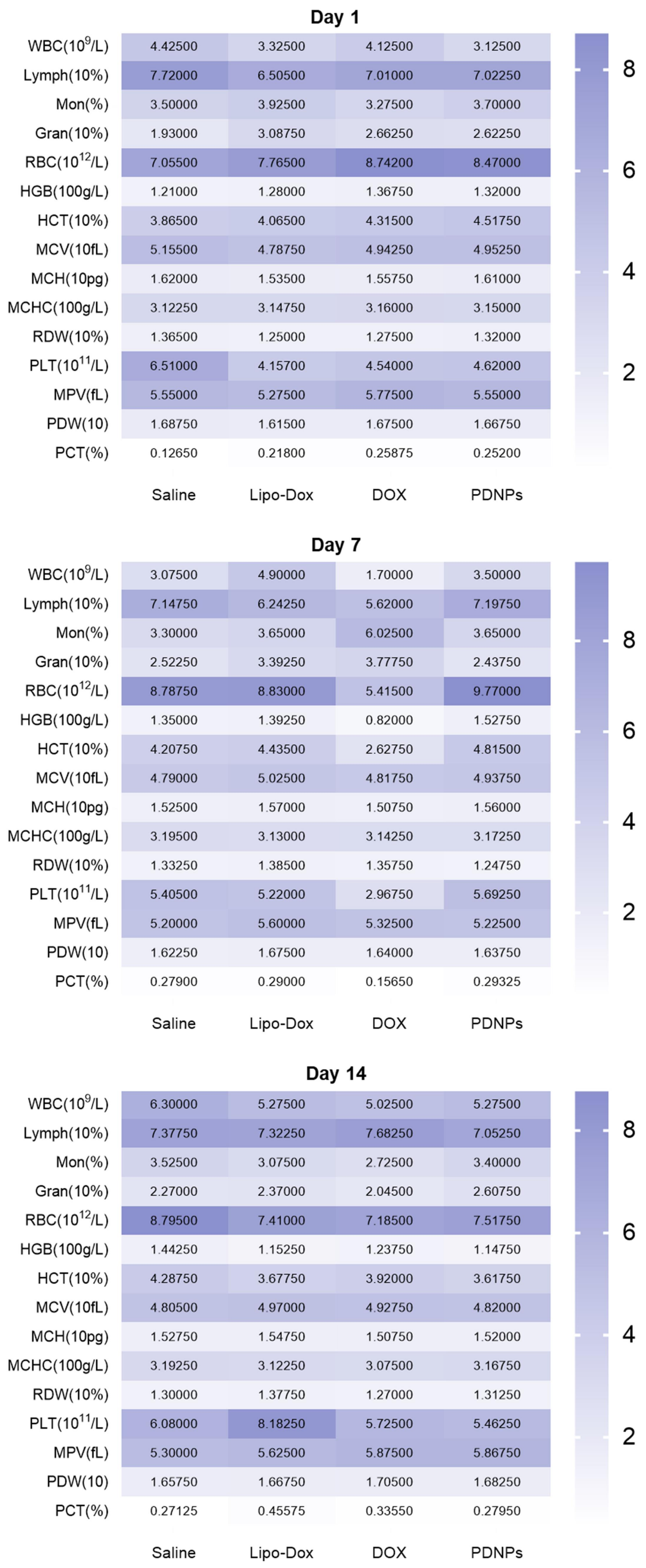

Figure S41. Blood routine examination on days 1, 7, and 14 after different treatments (Data presented as mean ± SD, n = 3).

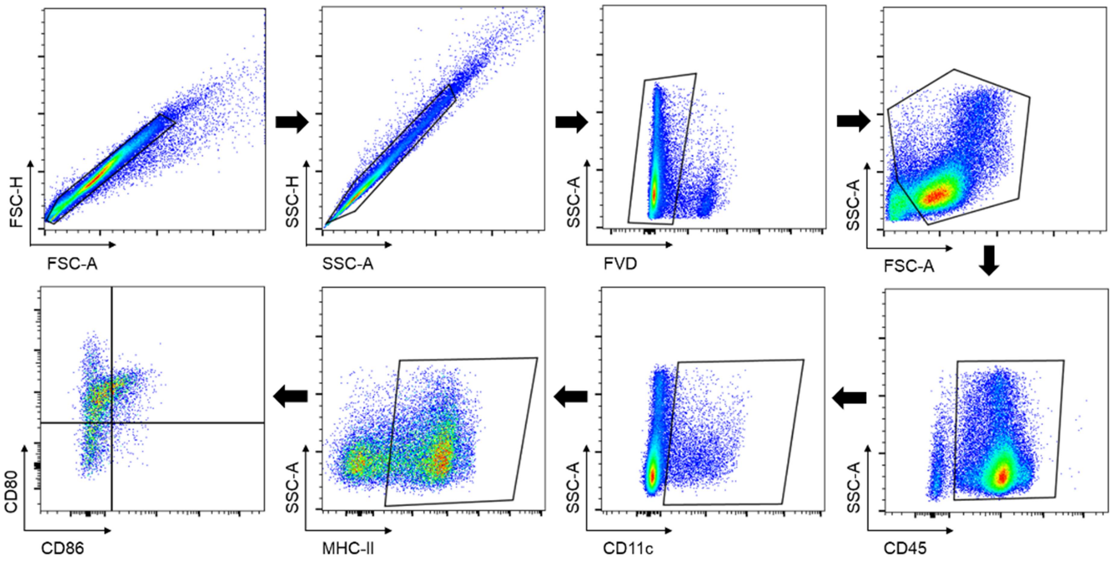

Figure S42. Dendritic cells gating strategy in flow cytometry analysis.

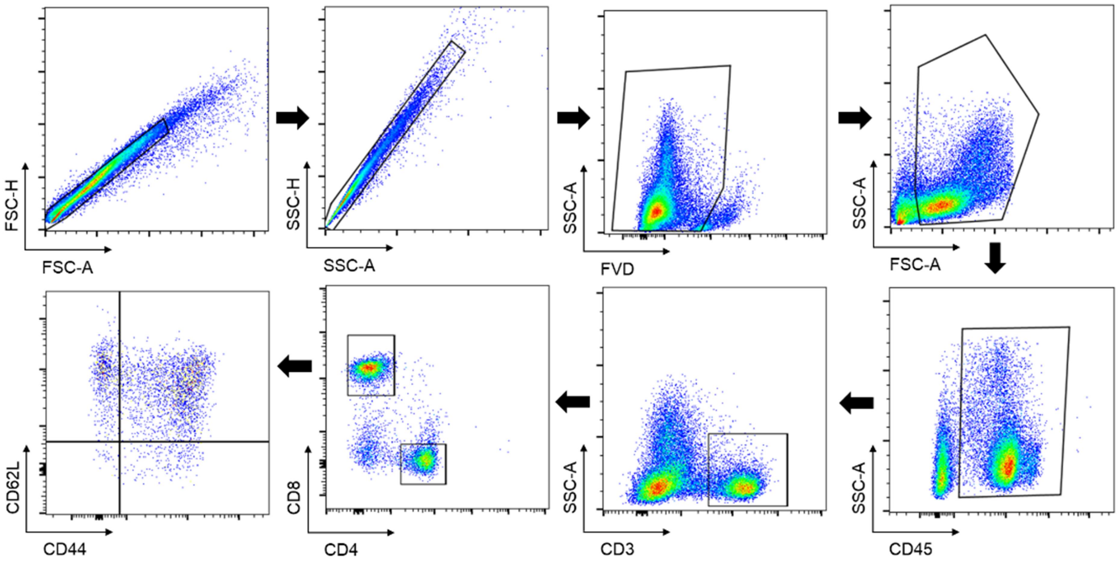

Figure S43. T cells gating strategy in flow cytometry analysis.

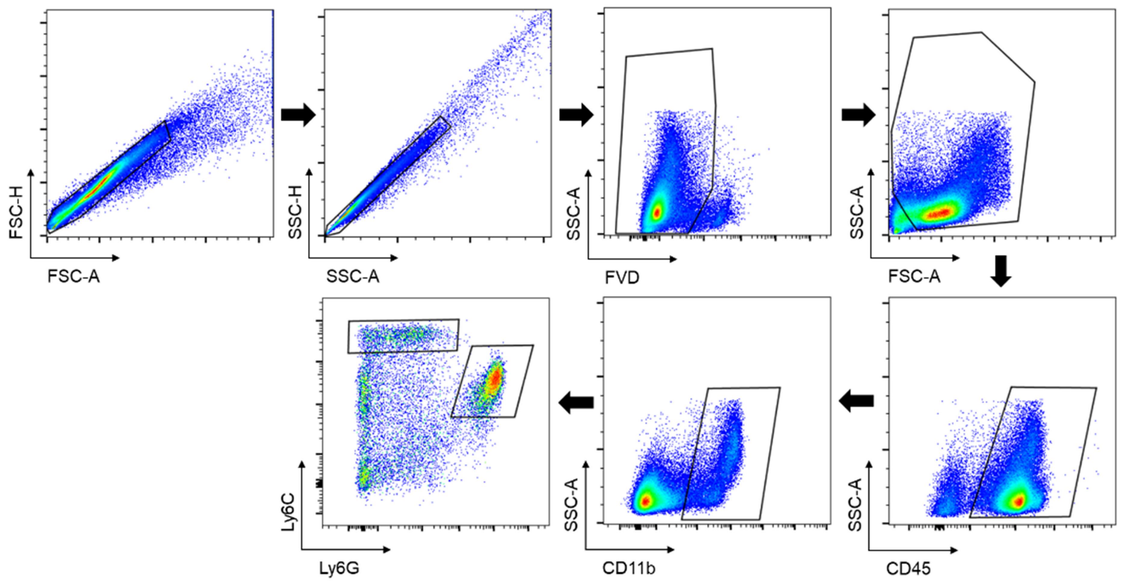

Figure S44. MDSCs gating strategy in flow cytometry analysis.

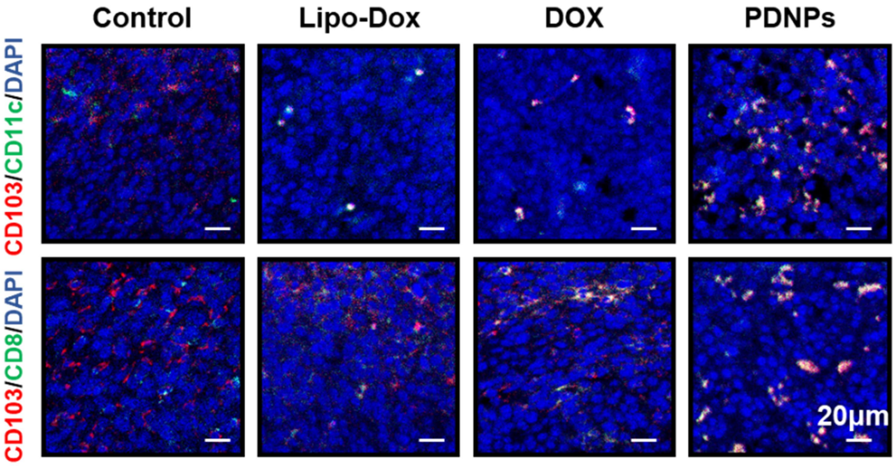

Figure S45. The representative immunofluorescence staining of infiltrating $CD103^{+}$ DCs and $CD103^{+}$ T cells in CT26 tumours collected from mice after treatments.

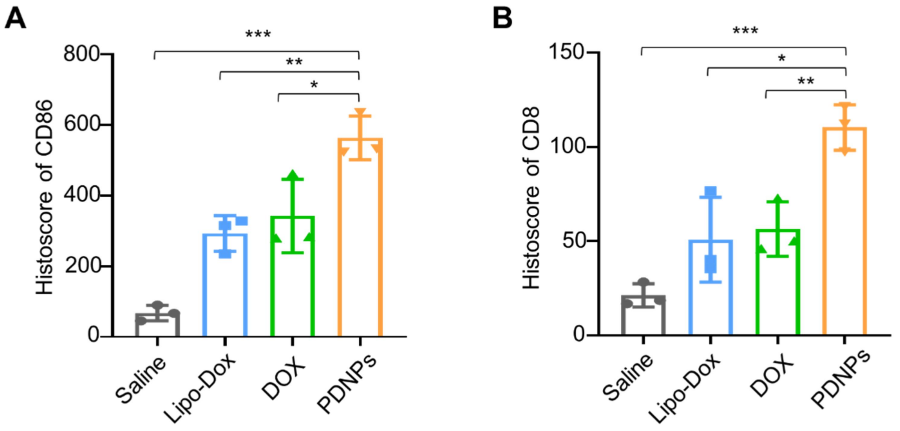

Figure S46. Quantitation of immunohistochemical of (A) CD86 and (B) CD8 in CT26 tumour tissues (Data presented as mean ± SD, n = 3, *P*-values are calculated using one-way ANOVA, **P* < 0.05, ***P* < 0.01, ****P* < 0.001).

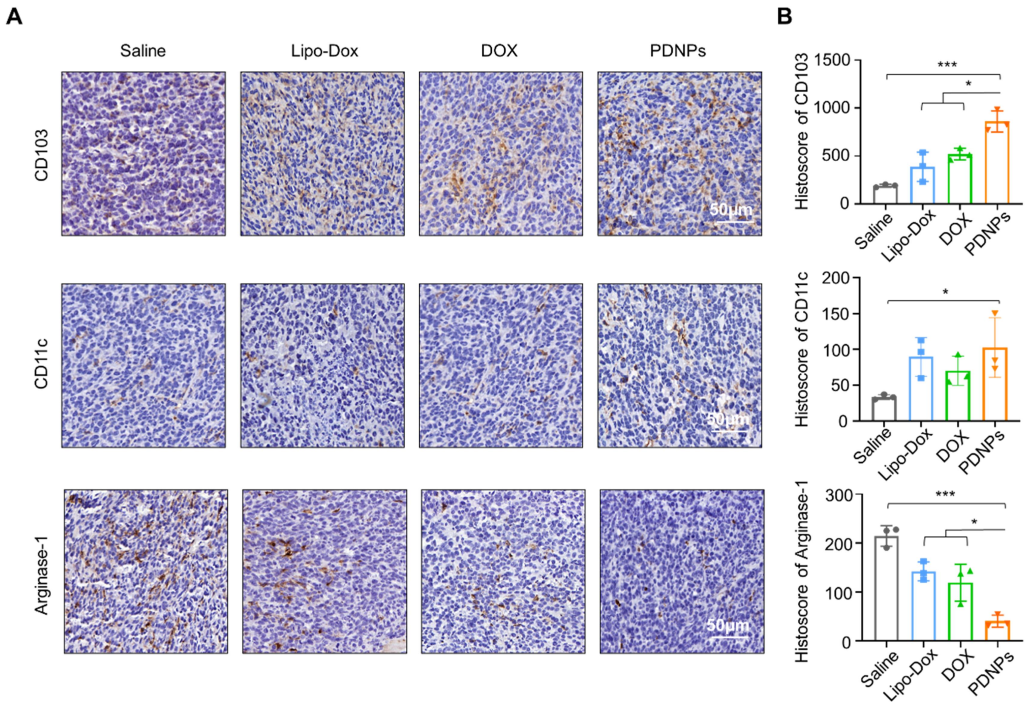

Figure S47. (A) Immunohistochemical staining of CD103, CD11c and Arginase-1 and (B) the corresponding quantitation in CT26 tumours collected from mice after treatments (Data presented as mean ± SD, n = 3, *P*-values are calculated using one-way ANOVA, **P* < 0.05, ****P* < 0.001).

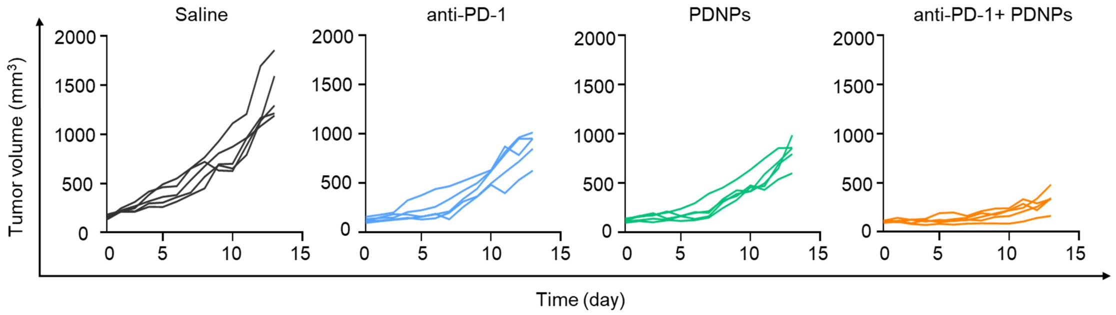

Figure S48. The individual tumour growth curves of CT26 tumours treated with saline, anti-PD-1, PDNPs and anti-PD-1 + PDNPs (Data presented as mean ± SD, n = 5).

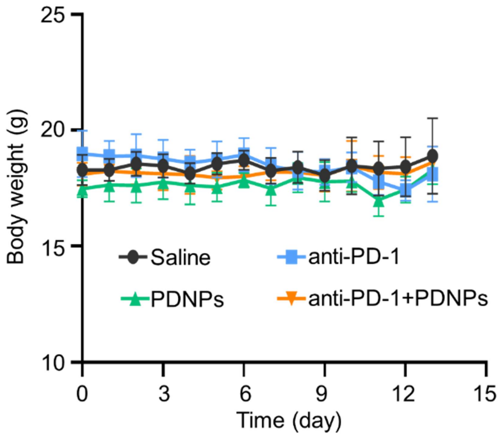

Figure S49. Body weight change of the CT26 tumour-bearing mice measured during the combination therapy (Data presented as mean ± SD, n = 5).

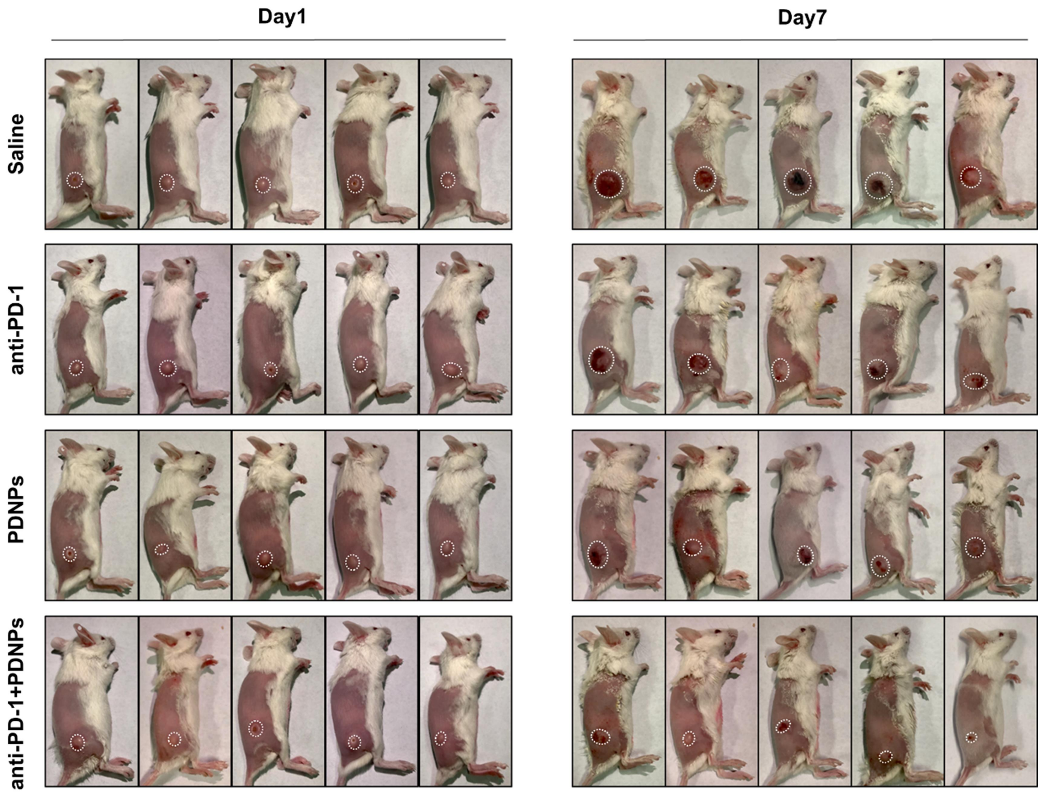

Figure S50. Photographs of CT26 tumour-bearing mice on days 1 and 7 during the combination therapy (Data presented as mean ± SD, n = 5).

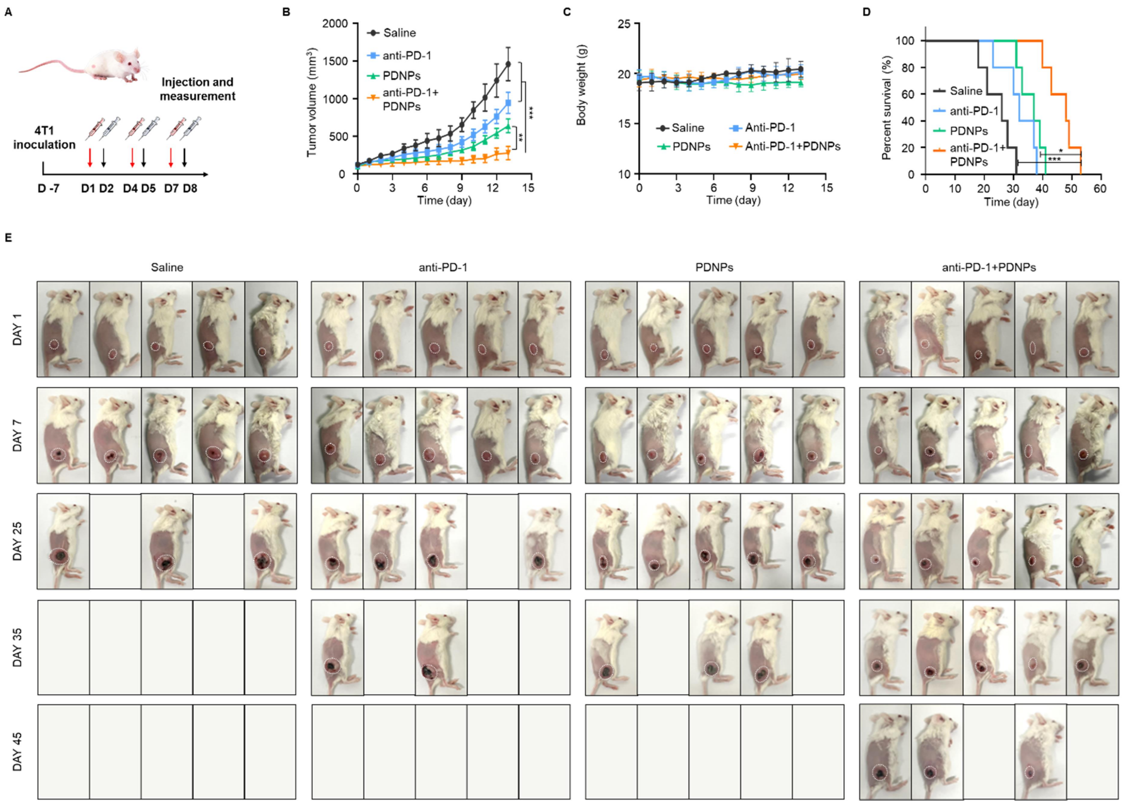

Figure S51. (A) Therapeutic strategy of PDNPs combined with anti-PD-1 therapy in 4T1-bearing mice (Red arrows denote PDNPs administration; Black arrows denote anti-PD-1 administration). (B) Growth curves of the combination therapy upon different treatments. (C) Body weight change of the 4T1 tumour-bearing mice measured during the combination therapy. (D) Survival curves of the combination therapy upon different treatments. (E) Bright field imaging of 4T1-bearing mice during the experiment. White circles denote the tumour region (Data presented as mean ± SD, n = 5, *P*-values are calculated using one-way ANOVA, *$P < 0.05$, **$P < 0.01$, ***$P < 0.001$).
